# Polyphenols of the Inuleae-Inulinae and Their Biological Activities: A Review

**DOI:** 10.3390/molecules29092014

**Published:** 2024-04-27

**Authors:** Janusz Malarz, Klaudia Michalska, Anna Stojakowska

**Affiliations:** Maj Institute of Pharmacology, Polish Academy of Sciences, Smętna Street 12, 31-343 Kraków, Poland; malarzj@if-pan.krakow.pl (J.M.); klaudiaz@if-pan.krakow.pl (K.M.)

**Keywords:** *Blumea*, chalcone, coumarin, *Dittrichia*, flavanone, flavone, hydroxycinnamate, *Inula*, lignan, *Pulicaria*

## Abstract

Polyphenols are ubiquitous plant metabolites that demonstrate biological activities essential to plant–environment interactions. They are of interest to plant food consumers, as well as to the food, pharmaceutical and cosmetic industry. The class of the plant metabolites comprises both widespread (chlorogenic acids, luteolin, quercetin) and unique compounds of diverse chemical structures but of the common biosynthetic origin. Polyphenols next to sesquiterpenoids are regarded as the major class of the Inuleae-Inulinae metabolites responsible for the pharmacological activity of medicinal plants from the subtribe (*Blumea* spp., *Dittrichia* spp., *Inula* spp., *Pulicaria* spp. and others). Recent decades have brought a rapid development of molecular and analytical techniques which resulted in better understanding of the taxonomic relationships within the Inuleae tribe and in a plethora of data concerning the chemical constituents of the Inuleae-Inulinae. The current taxonomical classification has introduced changes in the well-established botanical names and rearranged the genera based on molecular plant genetic studies. The newly created chemical data together with the earlier phytochemical studies may provide some complementary information on biochemical relationships within the subtribe. Moreover, they may at least partly explain pharmacological activities of the plant preparations traditionally used in therapy. The current review aimed to systematize the knowledge on the polyphenols of the Inulae-Inulinae.

## 1. Introduction

The Inuleae-Inulinae subtribe of the Asteraceae encompasses 28 genera and over 400 species of flowering plants. Majority of the species are native to Africa, Asia and the Mediterranean Basin [1]. The biggest genera of the tribe (*Blumea*, *Inula* and *Pulicaria*) that inhabit mainly Africa and Asia (*Blumea*), or Asia, Africa and Europe (*Inula*, *Pulicaria*) have been extensively chemically and pharmacologically studied due to their long history of use as traditional medicines, spices and insecticides [2,3,4,5,6,7,8].

Terpenoids (especially sesquiterpene lactones) and flavonoids are regarded as the active constituents responsible for the numerous biological effects exerted by the preparations from the plants of the Inuleae-Inulinae. A recent review on the cytotoxic activity of sesquiterpenoids and flavonoids from the selected genera of the Asteraceae described the cytotoxic effects demonstrated by the metabolites synthesized by *Blumea* spp., *Carpesium* spp. and *Inula* spp. [9] and emphasized the potent activity of sesquiterpenoids against the cancer cells in vitro. Except for the cytotoxic or antiproliferative action, the preparations from the plants of the subtribe are often investigated for their anti-inflammatory [10,11,12], antidiabetic [13,14,15], antilipidemic [16,17,18], vasorelaxant/antihypertensive [19,20], anti-oxidative stress [21,22,23] and hepatoprotective [24,25,26] activities in vivo. Recently, their efficacy in the prevention of depression-like behavior in rodents has been also explored [27,28]. The results of the studies have suggested that the polyphenolic metabolites are, at least in part, responsible for the observed pharmacological effects.

Flavonoids are the largest and the most extensively studied class of polyphenolics produced by the plants of the Inuleae-Inulinae. Together with hydroxycinnamates and hydroxybenzoates, they are the most frequently reported constituents of the plant infusions and alcoholic extracts. Their structural classification and biosynthesis have been recently summarized in a review paper by Chen and coworkers [29]. Other classes of polyphenolic metabolites from the Inuleae-Inulinae plants, including flavonolignans, lignans, coumarins and other compounds, have been found and characterized much less frequently.

Taxonomic investigations supported by the results of DNA sequencing caused the recent systematic rearrangements within the Inuleae tribe and, consequently, have led to some nomenclatural changes [30,31]. In the current review, the plant names that have the “accepted” status in the WFO database [32] are used. They are not always in accordance with the names commonly found in the literature. A list of the traditionally used plant binominal names and their counterparts that complies with the current classification is shown in Table 1.

The present review is based on the experimental results concerning the isolation and description of polyphenolic constituents from the Inuleae-Inulinae plants, qualitative analysis of the plant extracts using the hyphenated analytical techniques and pharmacological in vitro and in vivo studies on isolated polyphenolic plant metabolites that were published before January 2024 in the journals covered by two databases: Web of Science and Scopus. The majority of the papers that dealt with the polar (aqueous or alcoholic) extracts from the Inulae-Inulinae plants described their in vitro antioxidant and radical scavenging effects with the use of simple colorimetric assays. A large part of the literature contains either very limited or no chemical data on polyphenolic constituents of the investigated plant material. The chemical examination of the plant preparations is often limited to the ”total phenolics”, ”total tannins” and ”total flavonoids” assessments that are based on simple but rough spectrophotometric methods. This part of the literature is not covered by the current review.

## 2. Polyphenolic Metabolites of Inuleae-Inulinae

Flavonoids and derivatives of hydroxycinnamic acids are two classes of polyphenolic compounds that are common in Inuleae-Inulinae. They are often identified as the active constituents of plant extracts used in the traditional medicine. The most popular representatives of the group of plant constituents (quercetin, kaempferol, luteolin, chlorogenic acids) are widespread in the plant kingdom and are ubiquitous components of the human diet. Their putative role in the prevention of neurodegenerative and lifestyle diseases is still debatable but has found support in recent research [33,34,35] including clinical trials [36]. A substantial increase in a number of publications devoted to polyphenolics of Inuleae-Inulinae has taken place in the last decade. Application of the modern hyphenated analytical techniques speeded up the process of revealing the polyphenolic constituents present in the formerly uninvestigated or poorly described plant species. The techniques, however, have their limitations. Except for the well-studied plant metabolites of known mass fragmentation patterns, compound identification using different variants of high-performance liquid chromatography–mass spectrometry (HPLC-MS) is often incomplete (or doubtful). Likewise, the results obtained with simple chromatographic analytical techniques, like thin-layer chromatography (TLC) or HPLC with single wavelength detection, should be treated with caution, unless they are properly verified.

**Figure 1:**
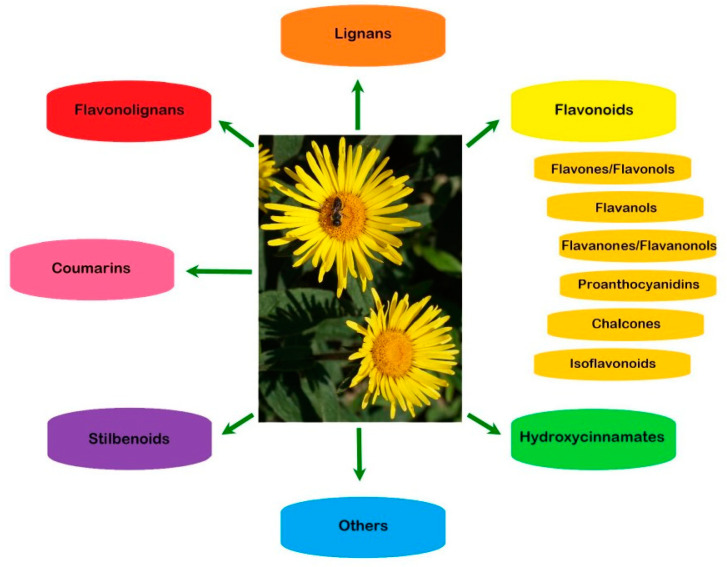
Polyphenolic constituents of the Inuleae-Inulinae.

In addition to flavonoids of different structural types and hydroxycinnamic acid derivatives (with the best-known chlorogenic acids) the plants of the Inuleae-Inulinae subtribe accumulate flavonolignans, lignans, stilbenoids, coumarins and other phenolic constituents (see Figure 1) which, although not abundant, may contribute to the biological activity of the parent plant. 

### 2.1. Structural Diversity of Flavonoids

Data in the literature on the flavonoids from the Inulae sensu lato, published until the beginning of the current century, have been summarized by Bohm and coworkers [37] in Chapter 12 of their comprehensive work: “Flavonoids of the Sunflower Family (Asteraceae)”. Since then, however, a large amount of data has been published and some changes in the taxonomic classification within the Inuleae tribe have been introduced. Flavonoids, next to sesquiterpenoids, have been the most often studied metabolites of the plants included in the tribe. Their structural diversity, particularly noticeable in such genera as *Blumea* and *Dittrichia*, along with their occurrence and distribution in the species of Inuleae-Inulinae has been summarized in Table 2, Table 3, Table 4 and Table 5. Dihydroderivatives of flavones (flavanones: naringenin, eriodictyol, hesperetin) and flavonols (flavanonols: taxifolin, aromadendrin), as well as the flavonoids oxygenated at C-6 of the A ring, seem to be characteristic of the described plant genera. The presence of multiple polymethoxylated flavonols, derivatives of quercetin and quercetagetin in the plants of the subtribe has also been frequently reported. 

#### 2.1.1. Flavones of the Inuleae-Inulinae

Apigenin and luteolin derivatives are the most frequently detected flavones in the plants of the subtribe. Chrysin, the flavone with the unsubstituted B ring and flavones with the substituents at C-3′, C-4′ and C-5′ (like tricin) are much less common (Table 2).

**Table 2:** Flavones of the Inuleae-Inulinae.

Trivial Name of the Compound	Substitution Pattern	Plant Species	Reference
Chrysin (CID: 5281607) Synonyms: Chrysine; Crysin	5,7-Dihydroxyflavone	*Chiliadenus glutinosus* *Inula helenium*; *I. inuloides*	[38] [39,40]
Chrysin 5-methyl ether (CID: 5490127)	7-Hydroxy-5-methoxyflavone	*Rhanterium adpressum*	[41]
(CID: 5282073)	7,4′-Dihydroxyflavone	*Inula salsoloides*	[42]
Apigenin (CID: 5280443) Synonyms: Versulin; Apigenol; Chamomile; Spigenin	5,7,4′-Trihydroxyflavone	*Asteriscus aquaticus*; *A. graveolens* *Blumea riparia* *Dittrichia viscosa* *Duhaldea cappa* *Inula anatolica*; *I. aucheriana*; *I. discoidea*; *I. inuloides*; *I. japonica*; *I. peacockiana*; *I. salsoloides*; *I. sarana*; *I. sechmenii*; *I. thapsoides*; *I. viscidula* *Pallenis hierochuntica*; *P. spinosa* *Pentanema aschersonianum*; *P. britannicum*; *P. ensifolium*; *P. mariae*; *P. oculus-christi*; *P. salicinum*; *P. spiraeifolium* *Rhanterium suaveolens* *Telekia speciosa*	[43,44] [45] [46,47] [48,49] [40,42,50,51,52,53] [54,55] [40,56,57,58,59] [60,61] [62]
Apigenin 7-*O*-glucoside (CID: 5280704) Synonyms: Apigetrin; Cosmosiin		*Blumea riparia* *Carpesium faberi* *Chrysophthalmum montanum* *Duhaldea cappa*; *D. cuspidata* *Inula anatolica*; *I. aucheriana*; *I. discoidea*; *I. inuloides*; *I. peacockiana*; *I. rhizocephala*; *I. royleana*; *I. sechmenii*; *I. stewartii*; *I. thapsoides*; *I. viscidula* *Pallenis hierochuntica* *Pentanema britannicum*; *P. mariae*; *P. oculus-christi* *Pulicaria undulata* *Rhanterium suaveolens* *Telekia speciosa* *Vicoa divaricata*; *Vicoa indica*	[45] [63] [64] [65,66] [40,65] [54] [40] [67] [60] [62] [65]
Apigenin 5-*O*-glucoside (CID: 14730805) Synonym: Salipurpin		*Duhaldea cappa*	[68]
Apigenin glucoside		*Dittrichia viscosa* *Pallenis spinosa*	[69] [55]
Apigenin glucoside malonate		*Dittrichia viscosa*	[70]
Apigenin 7-*O*-glucuronide (CID: 5319484) Synonym: Scutellarin A		*Inula japonica*	[71]
Apigenin *O*-hexuronide		*Telekia speciosa*	[62]
Apigenin dihexoside		*Pulicaria vulgaris*	[72]
Apigenin 8-*C*-glucoside (CID: 5280441) Synonym: Vitexin		*Asteriscus graveolens* *Duhaldea cappa*; *D. cuspidata*; *D. eupatorioides* *Limbarda crithmoides*	[44] [65] [73]
Apigenin 8-*C*-rhamnosylglucosyl		*Inula clarkei*; *I. koelzii*; *I. obtusifolia*; *I. rhizocephala*; *I. royleana*	[65]
Apigenin 6-*C*-glucoside (CID: 162350) Synonyms: Isovitexin; Saponaretin; Homovitexin		*Asteriscus graveolens* *Duhaldea cappa*; *D. cuspidata*; *D. nervosa*	[44] [65,74]
Apigenin 6,8-di-*C*-glucoside (CID: 3084407) Synonyms: Vicenin-2; Violantin		*Chiliadenus glutinosus* *Iphiona mucronata.*	[75] [76]
Apigenin 6-*C*-pentoside-8-*C*-hexoside		*Iphiona mucronata*	[76]
Apigenin 7-methyl ether (CID: 5281617) Synonym: Genkwanin	5,4′-Dihydroxy-7-methoxyflavone	*Asteriscus aquaticus* *Dittrichia viscosa*	[43] [46,77]
Apigenin 4′-methyl ether (CID: 5280442) Synonym: Acacetin	5,7-Dihydroxy-4′-methoxyflavone	*Duhaldea cappa* *Inula anatolica*; *I. peacockiana*; *I. salsoloides*; *I. sechmenii* *Pentanema oculus-christi* *Telekia speciosa*	[78] [40,42] [40] [62]
Apigenin 7,4′-dimethyl ether (CID: 5281601)	5-Hydroxy-7,4′-dimethoxyflavone	*Duhaldea nervosa*	[79]
6-Hydroxyapigenin (CID: 5281697) Synonym: Scutellarein	5,6,7,4′-Tetrahydroxyflavone	*Anvillea garcinii* *Dittrichia graveolens* *Inula acuminata*; *I. japonica* *Pentanema britannicum*; *P. caspicum* *Pulicaria dysenterica*	[80] [65] [52,65] [65] [81]
Scutellarein 7-methyl ether (CID: 3084390) Synonym: Sorbifolin	5,6,4′-Trihydroxy-7-methoxyflavone	*Pulicaria armena*; *P. vulgaris*	[82,83,84]
Ladanein (CID: 3084066) Synonym: Scutellarein 7,4′-dimethyl ether	5,6-Dihydroxy-7,4′-dimethoxyflavone	*Pulicaria paludosa*; *P. vulgaris*	[84,85]
Hispidulin (CID: 5281628) Synonyms: Dinatin; Scutellarein 6-methyl ether; 6-Methoxyapigenin	5,7,4′-Trihydroxy-6-methoxyflavone	*Anvillea garcinii* subsp. *radiata* *Dittrichia graveolens*; *D. viscosa* *Inula sarana* *Iphiona grantioides*; *I. mucronata* *Pentanema aschersonianum*; *P. britannicum*; *P. germanicum*; *P. montanum*; *P. oculus-christi* *Pulicaria insignis*; *P. paludosa*; *P. vulgaris* *Telekia speciosa*	[86] [46,47,87] [53] [76,88] [56,57,89,90,91,92,93] [94,95] [62]
Hispidulin 7-sulfate (CID: 13831736)		*Iphiona scabra*	[96]
Hispidulin 7-*O*-glucoside (CID: 44258433)		*Pentanema britannicum*; *P. montanum*	[92,97]
Hispidulin hexoside		*Dittrichia viscosa*	[98]
Cirsimaritin (CID: 188323) Synonyms: Scrophulein; Skrofulein; 7-Methylcapillarisin; 6-Methoxygenkwanin	5,4′-Dihydroxy-6,7-dimethoxyflavone	*Dittrichia viscosa* *Inula sarana* *Pentanema britannicum*; *P. montanum* *Rhanterium adpressum*	[99] [53] [57,92] [41]
Cirsimaritin derivative		*Dittrichia viscosa*	[77]
Pectolinarigenin (CID: 5320438) Synonyms: Scutellarein 6,4′-dimethyl ether; 6-Methoxyacacetin	5,7-Dihydroxy-6,4′-dimethoxyflavone	*Blumea lacera*	[100]
Scutellarein dimethyl ether		*Pulicaria paludosa*	[95]
Salvigenin (CID: 161271) Synonym: Scutellarein 6,7,4′-trimethyl ether	5-Hydroxy-6,7,4′-trimethoxyflavone	*Iphiona grantioides*; *I. mucronata*; *I. scabra* *Pulicaria undulata*	[76,88,96] [101]
(CID: 44259724)	5,6-Dihydroxy-3,7-dimethoxyflavone	*Pentanema britannicum*	[102]
(CID 5322076)	5,7,2′-Trihydroxy-6-methoxyflavone	*Chiliadenus glutinosus*	[38]
Grantionin (CID: 14861188)	7-Hydroxy-6,3′,5′-trimethoxyflavone	*Iphiona grantioides*	[103]
Luteolin (CID: 5280445) Synonyms: Flacitran; Luteoline	5,7,3′,4′-Tetrahydroxyflavone	*Asteriscus aquaticus*; *A. graveolens* *Blumea aromatica*; *B. balsamifera*; *B. megacephala (Randeria)*; *B. riparia* *Carpesium faberi* *Chiliadenus candicans*; *C. glutinosus* *Dittrichia viscosa* *Duhaldea cappa*; *D. nervosa*; *D. wissmanniana* *Inula anatolica*; *I. aucheriana*; *I. discoidea*; *I grandiflora*; *I helenium*; *I. inuloides*; *I. japonica*; *I. montbretiana*; *I. peacockiana*; *I. salsoloides*; *I. sarana*; *I. sechmenii*; *I. thapsoides*; *I. viscidula* *Pallenis hierochuntica* *Pentanema aschersonianum*; *P. britannicum*; *P. mariae*; *P. montanum*; *P. oculus-christi* *Pulicaria armena*; *P. gnaphalodes*; *P. incisa*; *P. salviifolia*; *P. schimperi*; *P. undulata*; *P. vulgaris* *Rhanterium suaveolens Desf* *Telekia speciosa (Schreb.) Baumg.*	[43,44] [45,104,105,106,107,108] [63] [38,109] [47,110] [48,111,112,113] [40,42,50,51,53,114,115,116] [54] [40,43,56,57,59,90] [82,117,118,119,120,121] [60,61] [62]
Luteolin 7-*O*-glucoside (CID: 5280637) Synonym: Cynaroside		*Asteriscus graveolens* *Blumea megacephala (Randeria)*; *Blumea riparia* *Buphthalmum salicifolium* *Carpesium cernuum* *Duhaldea cappa* *Inula anatolica*; *I. aucheriana*; *I. discoidea*; *I. helenium*; *I. inuloides*; *I. peacockiana*; *I. sarana*; *I. sechmenii*; *I. thapsoides*; *I. viscidula* *Pallenis hierochuntica* *Pentanema aschersonianum*; *P. britannicum*; *P. mariae*; *P. oculus-christi*; *P. orientale* *Rhanterium suaveolens* *Telekia speciosa*	[122] [45,123] [124] [125] [126] [39,40,53] [54] [40,56,65,127] [60,61] [62,128]
Caffeoyl cynaroside		*Blumea megacephala*; *B. riparia*	[123]
Luteolin malonyl-glucoside		*Pulicaria dysenterica*	[129]
Luteolin 7-*O*-glucuronide (CID: 5280601)		*Blumea megacephala (Randeria)*; *B. riparia*	[123]
Luteolin *O*-hexuronide		*Telekia speciosa*	[62]
Luteolin 7-*O*-glucuronide ethyl ester		*Duhaldea cappa*	[66]
Luteolin *O*-acetylhexoside		*Pulicaria undulata*	[130]
Luteolin 7-*O*-rutinoside (CID: 10461109) Synonym: Scolimoside		*Duhaldea cappa*	[66]
Luteolin 7-*O*-rutinoside/neohesperidoside		*Inula sarana*	[53]
Luteolin-3′-*O*-glucoside (CID: 12309350) Synonym: Dracocephaloside		*Duhaldea cappa*	[49]
Luteolin 4′-*O*-glucoside (CID: 5319116)		*Blumea sinuate*; *B. balsamifera* *Duhaldea cappa* *Pallenis hierochuntica*	[131,132] [68] [54]
Luteolin 8-*C*-glucoside (CID: 5281675) Synonym: Orientin		*Asteriscus graveolens*	[44]
Luteolin 6-*C*-glucoside (CID: 114776) Synonym: Isoorientin; Homoorientin		*Asteriscus graveolens* *Dittrichia viscosa* *Duhaldea cappa*; *D. cuspidata* *Inula clarkei*; *I. koelzii*; *I. racemosa*; *I. royleana*; *I. stewartii*	[44] [69] [65] [65]
Luteolin 7,4′-diglucoside (CID: 14769208)		*Pallenis hierochuntica*	[54]
Luteolin 7-methyl ether (CID: 5318214) Synonym: Hydroxygenkwanin		*Blumea balsamifera* *Duhaldea nervosa* *Pallenis hierochuntica*	[105,106,133] [112] [54]
Chrysoeriol (CID: 5280666) Synonyms: Luteolin 3′-methyl ether; 3′-Methoxyapigenin; 3′-O-Methylluteolin	5,7,4′-Trihydroxy-3′-methoxyflavone	*Blumea aromatica*; *B. balsamifera*; *B. megacephala (Randeria)*; *B. riparia* *Dittrichia viscosa* *Duhaldea cappa* *Inula japonica*; *I. sarana* *Pentanema aschersonianum*; *P. britannicum* *Pulicaria incisa* *Telekia speciosa*	[104,108,123,134] [135] [68] [53,116] [56,57] [25] [62]
Chrysoeriol 7-*O*-glucoside (CID: 11294177) Synonym: Thermopsoside		*Blumea megacephala*; *B. riparia*	[123]
Caffeoyl thermopsoside		*Blumea megacephala*; *B. riparia*	[123]
Chrysoeriol 4′-*O*-glucoside		*Duhaldea cappa*	[68]
Chrysoeriol *O*-hexoside		*Inula sarana* *Pulicaria undulata*	[53] [130]
Diosmetin (CID: 5281612) Synonym: Luteolin 4′-methyl ether	5,7,3′-Trihydroxy-4′-methoxyflavone	*Blumea balsamifera*; *B. megacephala* *Dittrichia viscosa* *Duhaldea cappa* *Inula japonica*	[108,136] [77] [78] [116]
Diosmetin 7-rhamnoglucoside CID: 5281613) Synonym: Diosmin		*Anvillea garcinii* subsp. *radiata*	[137]
Luteolin 5-methyl ether (CID: 13964550)		*Inula salsoloides*	[42]
Velutin (CID: 5464381) Synonym: Luteolin 7,3′-dimethyl ether	5,4′-Dihydroxy-7,3′-dimethoxyflavone	*Blumea aromatica*; *B. balsamifera*; *B. lacera*	[100,105,133,138]
Luteolin 7,3′,4′-trimethyl ether	5-Hydroxy-7,3′,4′-trimethoxyflavone	*Blumea aromatica* *Pulicaria salviifolia*	[104,138] [119]
(CID: 5487757)	5,7,3′,5′-Tetrahydroxyflavone	*Inula salsoloides*	[42]
8-Methoxyluteolin (CID: 5316843) Synonym: Onopordin	5,7,3′,4′-Tetrahydroxy-8-methoxyflavone	*Inula japonica*	[51]
6-Hydroxyluteolin (CID: 5281642)	5,6,7,3′,4′-Pentahydroxyflavone	*Anvillea garcinii* *Pulicaria paludosa*	[80] [95]
Hydroxyluteolin hexoside		*Dittrichia viscosa*	[139]
Pedalitin (CID: 31161) Synonym: 6-Hydroxyluteolin 7-methyl ether	5,6,3′,4′-Tetrahydroxy-7-methoxyflavone	*Pulicaria paludosa*	[95]
6-Hydroxyluteolin 7,3′-dimethyl ether (CID: 10359254)	5,6,4′-Trihydroxy-7,3′-dimethoxyflavone	*Blumea lacera* *Pulicaria vulgaris* *Vicoa indica*	[100] [84] [140,141]
6-Hydroxyluteolin 7,4′-dimethyl ether	5,6,3′-Trihydroxy-7,4′-dimethoxyflavone	*Pulicaria armena*	[82]
6-Hydroxyluteolin trimethyl ether		*Pulicaria paludosa*	[95]
6-Methoxyluteolin (CID: 5317284) Synonyms: Nepetin; Eupafolin	5,7,3′,4′-Tetrahydroxy-6-methoxyflavone	*Anvillea garcinii* subsp. *radiata* *Dittrichia viscosa* *Duhaldea nervosa* *Inula japonica*; *I. sarana* *Pentanema aschersonianum*; *P. britannicum*; *P. germanicum*; *P. montanum*; *P. salicinum* *Pulicaria insignis*; *P. paludosa* *Telekia speciosa*	[86] [47,77] [111] [51,53,116] [43,56,57,90,92] [94,95] [62]
6-Methoxyluteolin hexoside		*Anvillea garcinii* subsp. *radiata* *Telekia speciosa*	[142] [62]
6-Methoxyluteolin *O*-glucoside		*Anvillea garcinii* subsp. *radiata* *Inula sarana*	[142] [53]
6-Methoxyluteolin 7-*O*-glucoside (CID: 120742) Synonym: Nepitrin		*Blumea megacephala*; *B. riparia* *Inula japonica* *Pentanema aschersonianum*; *P. britannicum*; *P. montanum*	[45,123] [51] [56,92,97]
6-Methoxyluteolin *O*-rutinoside		*Inula sarana*	[53]
Jaceosidin (CID: 5379096) Synonym: 6-Methoxyluteolin 3′-methyl ether	5,7,4′-Trihydroxy-6,3′-dimethoxyflavone	*Anvillea garcinii* subsp. *radiata* *Dittrichia viscosa* *Inula sarana* *Pentanema germanicum*	[86,143] [70] [53] [57]
Jaceoside (CID: 11179379) Synonym: Jaceosidin 7-*O*-glucoside		*Pentanema montanum* *Telekia speciosa*	[92] [62]
Demethoxycentaureidin (CID: 5469524) Synonyms: Desmethoxycentaureidin; 6-Methoxyluteolin 4′-methyl ether	5,7,3′-Trihydroxy-6,4′-dimethoxyflavone	*Blumea lacera*	[100]
Cirsiliol (CID: 160237) Synonym: 6-Methoxyluteolin 7-methyl ether	5,3′,4′-Trihydroxy-6,7-dimethoxyflavone	*Dittrichia viscosa* *Inula sarana* *Pentanema britannicum*	[77] [53] [57]
Cirsiliol *O*-hexoside		*Inula sarana*	[53]
Cirsilineol (CID: 162464) Synonyms: Eupatrin; Fastigenin	5,4′-Dihydroxy-6,7,3′-trimethoxyflavone	*Blumea lacera*	[100,144]
Eupatilin (CID: 5273755) Synonym: 6-Methoxyluteolin 3′,4′-dimethyl ether	5,7-Dihydroxy-6,3′,4′-trimethoxyflavone	*Blumea lacera* *Inula sarana* *Pentanema britannicum* *Pulicaria dysenterica*	[100] [53] [57] [129]
Sinensetin (CID: 145659) Synonym: 6-Methoxyluteolin tetramethyl ether	5,6,7,3′,4′-Pentamethoxyflavone	*Pulicaria paludosa*; *P. sicula*	[95]
Xanthomicrol (CID: 73207)	5,4′-Dihydroxy-6,7,8-trimethoxyflavone	*Chiliadenus iphionoides*	[145,146]
Tricin (CID: 5281702)	5,7,4′-Trihydroxy-3′,5′-dimethoxyflavone	*Blumea megacephala*; *B. riparia* *Inula helenium* *Pallenis spinosa* *Pulicaria incisa*	[45,123] [147] [148] [25]
Tricin 7-*O*-glucoside (CID: 5322022)		*Blumea riparia* *Pallenis spinosa*	[45] [148,149]
Tricin 7-*O*-malonylglucoside		*Blumea megacephala*; *B. riparia*	[123]
Tricin 5-*O*-glucoside (CID: 49800176)		*Iphiona aucheri*; *I. grantioides* *Pallenis spinosa*	[65] [149]
Tricin *O*-hexoside		*Iphiona mucronata*	[76]
Feruloyl tricin		*Blumea megacephala*; *B. riparia*	[123]
Salcolin A (CID: 21575482) Synonym: Tricin 4′-O-(threo-beta-guaiacylglyceryl) ether		*Blumea megacephala*; *B. riparia*	[123]
Ageconyflavone C (CID: 44258535)	4′-Hydroxy-5,6,7,3′,5′-pentamethoxyflavone	*Blumea fistulosa*	[150]
CID: 185670	5,6,7,3′,4′,5′-Hexamethoxyflavone	*Blumea fistulosa*	[150]
Nobiletin (CID: 72344)	5,6,7,8,3′,4′-Hexamethoxyflavone	*Blumea fistulosa*	[150]
5′-Methoxynobiletin (CID: 72815)	5,6,7,8,3′,4′,5′-Heptamethoxyflavone	*Blumea fistulosa*	[150]
	5,6,7,8,5′-Pentamethoxy-3′,4′-methylenedioxyflavone	*Blumea fistulosa*	[150]
**Flavone dimers**			
Amentoflavone 7,4′,4′′′-trimethyl ether (CID: 5281696) Synonym: Sciadopitysin	Biflavon	*Blumea balsamifera*	[151]
3-*O*-7″-Biluteolin	Biflavon	*Blumea balsamifera*	[152]

**Figure 2:**
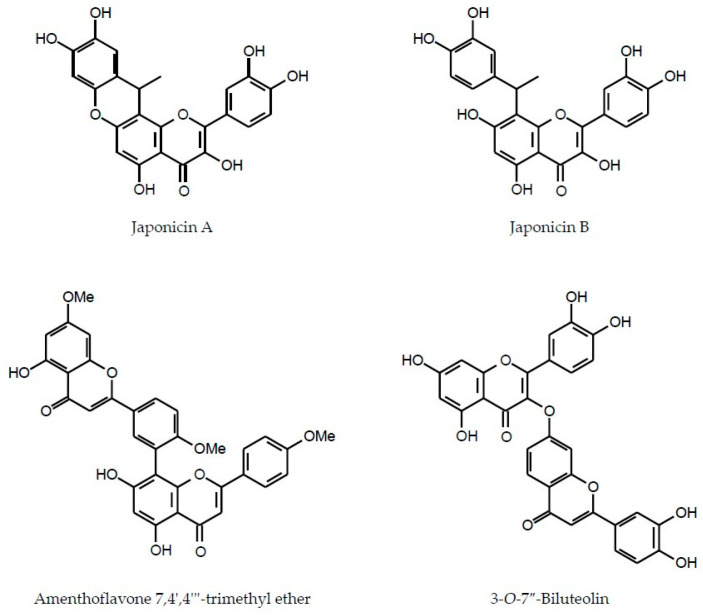
Structures of japonicins A and B from *Inula japonica* and biflavones from *Blumea balsamifera*.

As can be seen in the table above, flavone dimers amentoflavone 7,4′,4′′′-trimethyl ether and 3-*O*-7″-biluteolin (Figure 2) were found solely in *Blumea balsamifera* [151,152]. Plants of the genera *Asteriscus*, *Chiliadenus*, *Dittrichia*, *Duhaldea*, *Inula*, *Iphiona* and *Limbarda* were found to synthesize flavone C-glycosides [44,65,69,73,74,75,76]. Nobiletin and its derivatives from *B. fistulosa* [150] and xanthomicrol from *C. iphionoides* [145,146] are the examples of the rare C-8 methoxylated flavones.

#### 2.1.2. Flavonols of the Inuleae-Inulinae

Flavonols are the most numerous subclass of flavonoids synthesized by the Inuleae-Inulinae. Except for the most frequently found kaempferol and quercetin derivatives, the methyl ethers and glycosides of 6-hydroxykaempferol and 6-hydroxyquercetin (quercetagetin) were often isolated from the plants of the subtribe (see Table 3).

**Table 3:** Flavonols from the Inuleae-Inulinae.

Trivial Name of the Compound	Substitution Pattern	Plant Species	Reference
Galangin (CID: 5281616)	3,5,7-Trihydroxyflavone	*Limbarda crithmoides*	[153]
Kaempferol (CID: 5280863) Synonyms: Robigenin; Kaempherol; Kempferol; Populnetin; Rhamnolutein; Trifolitin	3,5,7,4′-Tetrahydroxyflavone	*Asteriscus aquaticus* *Blumea aromatica*; *B. lacera*; *B. sinuata* *Chiliadenus glutinosus*; *C. iphionoides* *Chrysophthalmum montanum* *Dittrichia graveolens*; *D. viscosa* *Duhaldea nervosa* *Inula anatolica*; *I. aucheriana*; *I. discoidea*; *I. helenium*; *I. inuloides*; *I. japonica*; *I. peacockiana*; *I. salsoloides*; *I. sarana*; *I. sechmenii*; *I. thapsoides*; *I. viscidula* *Pallenis spinosa* *Pentanema britannicum*; *P. mariae*; *P. oculus-christi* *Pulicaria arabica*; *P. dysenterica*; *P. gnaphalodes*; *P. incisa*; *P. jaubertii*; *P. vulgaris* *Rhanterium suaveolens* *Telekia speciosa*	[43] [138,154] [11,155] [64] [87,99] [111] [40,42,53,115,116,156,157] [55,149] [40,59,97] [57,117,121,130,158,159,160,161] [61] [62]
Kaempferol 3-*O*-glucoside (CID: 5282102) Synonym: Astragalin		*Anvillea garcinii* *Asteriscus graveolens* *Buphthalmum salicifolium*; *B. speciosissimum* *Carpesium cernuum* *Chiliadenus glutinosus* *Inula anatolica*; *I. aucheriana*; *I. discoidea*; *I. inuloides*; *I. peacockiana*; *I. sarana*; *I. sechmenii*; *I. thapsoides*; *I. viscidula* *Pentanema britannicum*; *P. mariae* *Pulicaria jaubertii*; *P. undulata*	[162] [122] [124,163] [125] [38] [40,53] [40,97] [160,164]
Kaempferol-3-*O*-(6″-*O*-acetyl)-glucoside (CID: 10435673) Synonyms: 6″-*O*-Acetylastragalin; Kaempferol 3-*O*-acetyl-glucoside		*Chiliadenus montanus*	[165,166]
Kaempferol 3-*O*-galactoside (CID: 5282149) Synonyms: Trifolin; Trifolioside		*Asteriscus graveolens* *Pulicaria dysenterica*; *P. incisa*; *P. schimperi*	[122] [118,167,168]
Kaempferol 3-*O*-glucuronide (CID: 5318759)		*Chiliadenus glutinosus* *Dittrichia viscosa* *Telekia speciosa*	[169] [170] [62]
Kaempferol 7-*O*-glucoside (CID: 10095180)		*Anvillea garcinii* *Asteriscus graveolens*	[162,171] [122]
Kaempferol 3-*O*-pentoside		*Dittrichia viscosa*	[172]
Kaempferol *O*-pentoside		*Dittrichia viscosa*	[173]
Kaempferol 3-*O*-hexoside		*Iphiona mucronata*	[76]
Kaempferol *O*-hexoside		*Dittrichia viscosa*	[170,173]
Kaempferol *O*-(acetyl)-hexoside		*Dittrichia viscosa*	[172]
Kaempferol 3-*O*-(caffeoyl)-hexoside		*Dittrichia viscosa*	[172]
Kaempferol *O*-(*p*-coumaroyl)-hexoside		*Dittrichia viscosa*	[172]
Kaempferol *O*-(feruloyl)-hexoside		*Dittrichia viscosa*	[172]
Kaempferol 7-*O*-hexoside		*Dittrichia viscosa*	[172]
Kaempferol *O*-*p*-coumaroyl-*O*-hexoside		*Dittrichia viscosa*	[172]
Kaempferol 3-*O*-rutinoside (CID: 5318767) Synonyms: Nicotiflorin; Nictoflorin; Nicotifloroside		*Anvillea garcinii* *Carpesium cernuum* *Chiliadenus glutinosus* *Dittrichia viscosa* *Duhaldea nervosa* *Inula anatolica*; *I. aucheriana*; *I. discoidea*; *I. inuloides*; *I. peacockiana*; *I. sarana*; *I. sechmenii*; *I. thapsoides*; *I. viscidula* *Pentanema britannicum*; *P. mariae* *Pulicaria undulata* *Rhanterium suaveolens*	[171] [125] [75] [99] [111] [40,53] [40] [120] [60]
Kaempferol 7-*O*-neohesperidoside		*Duhaldea nervosa*	[74]
Kaempferol 7-*O*-dipentoside		*Inula helenium*; *I. racemosa*	[21,174]
Kaempferol 3-*O*-rutinoside 7-*O*-glucuronide		*Dittrichia graveolens* *Inula clarkei*; *I. obtusifolia*	[65]
Kaempferol 3-*O*-sophoroside 7-*O*-rhamnoside		*Pentanema orientale*	[65]
Kaempferol 3-*O*-sophorotrioside 7-*O*-rhamnoside		*Dittrichia graveolens* *Inula acuminata*; *I. koelzii*; *I. racemosa*; *I. royleana*; *I. stewartii* *Pentanema caspicum* *Vicoa glanduligera*; *V. divaricata*	[65] [65] [65] [65]
Kaempferol 3-*O*-lathyroside 7-*O*-rhamnoside		*Pentanema britannicum*; *P. orientale*	[65]
Isokaempferide (CID: 5280862) Synonym: Kaempferol 3-methyl ether	5,7,4′-Trihydroxy-3-methoxyflavone	*Allagopappus viscosissimus* *Chiliadenus candicans*; *C. iphionoides* *Dittrichia graveolens*; *D. viscosa* *Inula hookeri* *Pallenis spinosa* *Pulicaria arabica*; *P. dysenterica*; *P. incisa*; *P. insignis*; *P. jaubertii*; *P. undulata*	[175] [109,176,177] [46,47,87] [178] [149] [57,94,158,159,168,179]
Kaempferol 3-methyl ether 6-*O*-glucoside		*Pulicaria dysenterica*	[81]
Kaempferide (CID: 5281666) Synonym: Kaempferol 4′-methyl ether	3,5,7-Trihydroxy-4′-methoxyflavone	*Blumea balsamifera* *Dittrichia viscosa* *Pentanema conyzae*	[134] [180] [43]
Rhamnocitrin (CID: 5320946) Synonym: Kaempferol 7-methyl ether	3,5,4′-Trihydroxy-7-methoxyflavone	*Blumea riparia* *Dittrichia viscosa*	[181] [46,47]
Kumatakenin (CID: 5318869) Synonym: Kaempferol 3,7-dimethyl ether		*Chiliadenus iphionoides* *Pulicaria arabica*; *P. jaubertii*	[145,146,176] [158,159]
6-Hydroxykaempferol (CID: 5281638)	3,5,6,7,4′-Pentahydroxyflavone		
6-Hydroxykaempferol 3-sulfate		*Pentanema britannicum*	[182]
6-Hydroxykaempferol 7-*O*-glucoside (CID: 44259740)		*Buphthalmum salicifolium*; *B. speciosissimum*	[124,163]
6-Hydroxykaempferol 3-methyl ether 6-*O*-glucoside (CID: 44259742)		*Pulicaria undulata*	[183]
6-Hydroxykaempferol 3-methyl ether 6-*O*-glucosyl-(1->6)-glucoside		*Pulicaria undulata*	[183]
6-Hydroxykaempferol 3,7-dimethyl ether (CID: 13983730)	5,6,4′-Trihydroxy-3,7-dimethoxyflavone	*Inula grandis* *Pentanema montanum* *Pulicaria dysenterica*; *P. inuloides*	[184] [90,92] [57,81,185,186]
6-Hydroxykaempferol 7,4′-dimethyl ether	3,5,6-Trihydroxy-7,4′-dimethoxyflavone	*Pulicaria dysenterica*	[187]
6-Hydroxykaempferol 3,5,7-trimethyl ether (CID: 14376219)	6,4′-Dihydroxy-3,5,7-trimethoxyflavone	*Chiliadenus candicans*	[109]
6-Hydroxykaempferol 3,7,4′-trimethyl ether (CID: 10043097) Synonym: Tanetin	5,6-Dihydroxy-3,7,4′-trimethoxyflavone	*Pentanema conyzae* *Pulicaria dysenterica*; *P. odora*	[43] [95,187]
Hydroxykaempferol trimethyl ether		*Pulicaria vulgaris*	[72]
Hydroxykaempferol tetramethyl ether		*Pulicaria vulgaris*	[72]
6-Methoxykaempferol (CID: 5377945)	3,5,7,4′-Tetrahydroxy-6-methoxyflavone	*Dittrichia viscosa* *Inula sarana* *Pulicaria odora*; *P. undulata* *Telekia speciosa*	[47] [53] [95,183,188] [43,62]
6-Methoxykaempferol 3-*O*-glucoside (CID: 44259734)		*Anvillea garcinii* subsp. *radiata* *Blumea lacera* *Pulicaria undulata*	[86] [100] [183]
6-Methoxykaempferol 7-*O*-glucoside (CID: 44259747)		*Pulicaria odora*	[95]
6-Methoxykaempferol 3-*O*-galactoside (CID: 44259725)		*Anvillea garcinii*	[80]
6-Methoxykaempferol 3-*O*-rhamnoglucoside		*Anvillea garcinii*	[80]
6-Methoxykaempferol 3-*O*-galactoside 7-methyl ether		*Anvillea garcinii*	[80]
Eupalitin (CID: 5748611) Synonym: 6-Methoxykaempferol 7-methyl ether	3,5,4′-Trihydrox-6,7-dimethoxyflavone	*Pulicaria dysenterica*	[129]
6-Methoxykaempferol 4′-methyl ether (CID: 5459196) Synonym: Betuletol	3,5,7-Trihydroxy-6,4′-dimethoxyflavone	*Pentanema conyzae*	[43]
6-Methoxykaempferol 3-methyl ether (CID: 5352032) Synonym: 3,6-Dimethoxyapigenin	5,7,4′-Trihydroxy-3,6-dimethoxyflavone	*Chiliadenus candicans*; *C. iphionoides* *Dittrichia graveolens* *Pulicaria insignis*; *P. paludosa* *Telekia speciosa*	[109,176,177] [87] [85,94] [43]
6-Methoxykaempferol 3,7-dimethyl ether (CID: 5320462) Synonym: Penduletin	5,4′-Dihydroxy-3,6,7-trimethoxyflavone	*Chiliadenus candicans*; *C. montanus*; *C. iphionoides* *Duhaldea wissmanniana* *Pentanema spiraeifolium* *Pulicaria dysenterica*	[109,176,177,189,190] [113] [43] [81]
6-Methoxykaempferol 3,4′-dimethyl ether (CID: 5281695) Synonym: Santin	5,7-Dihydroxy-3,6,4′-trimethoxyflavone	*Pulicaria insignis*	[94]
6-Methoxykaempferol 7,4′-dimethyl ether (CID: 15560536) Synonym: Mikanin	3,5-Dihydroxy-6,7,4′-trimethoxyflavone	*Pentanema conyzae*	[43]
Mikanin 3-*O*-galactoside (CID: 44259729)		*Anvillea garcinii*	[80]
6-Methoxykaempferol 3,7,4′-trimethyl ether (CID: 5318355) Synonym: Penduletin 4′-methyl ether	5-Hydroxy-3,6,7,4′-tetramethoxyflavone	*Blumea malcolmii* *Iphiona scabra* *Pentanema conyzae* *Pulicaria odora*; *P. sicula*	[191] [96] [43] [43,95]
6-Methoxykaempferol 3,5,7-trimethyl ether (CID: 13983731)	4′-Hydroxy-3,5,6,7-tetramethoxyflavone	*Chiliadenus iphionoides*	[177]
6-Methoxykaempferol 3,5,7,4′-tetramethyl ether (CID: 521171)	3,5,6,7,4′-Pentamethoxyflavone	*Chiliadenus montanus*; *C. iphionoides* *Pulicaria odora*	[177,192] [95]
	3,6,8-Trihydroxy-7,4′-dimethoxyflavone	*Pulicaria paludosa*	[85]
(CID: 44258717)	3,5,2′-Trihydroxy-7,5′-dimethoxyflavone	*Blumea balsamifera*	[193]
Quercetin (CID: 5280343 Synonyms: Meletin; Sophoretin; Quercetine; Xanthaurine; Quercetol; Quertine	3,5,7,3′,4′-Pentahydroxyflavone	*Anvillea garcinii* subsp. *radiata* *Asteriscus graveolens* *Blumea aromatica*; *B. balsamifera*; *B. lacera*; *B. megacephala*; *B. riparia*; *B. sinuata* *Chiliadenus glutinosus*; *C. iphionoides*; *C. montanus* *Chrysophthalmum montanum* *Dittrichia graveolens*; *D. viscosa* *Duhaldea nervosa* *Inula anatolica*; *I. aucheriana*; *I. discoidea*; *I. grandiflora*; *I. helenium*; *I. inuloides*; *I. japonica*; *I. montbretiana*; *I. obtusifolia*; *I. peacockiana*; *I. racemosa*; *I. sarana*; *I. sechmenii*; *I. thapsoides*; *I. viscidula* *Limbarda crithmoides* *Pallenis hierochuntica*; *P. spinosa* *Pentanema britannicum*; *P. conyzae*; *P. mariae*; *P. oculus-christi* *Pulicaria arabica*; *P. armena*; *P. dysenterica*; *P. gnaphalodes*; *P. incisa*; *P. jaubertii*; *P. salviifolia*; *P. schimperi*; *P. sicula*; *P. undulata*; *P. vulgaris* *Rhanterium adpressum*; *R. suaveolens* *Telekia speciosa* *Vicoa glanduligera*	[194] [122] [104,105,106,107,108,154,195,196] [11,75,155,190,197] [64] [180,198,199,200] [201] [40,51,53,65,114,115,116,202,203,204] [205,206] [54,148,207] [40,43,58,127] [43,57,67,72,82,117,118,119,121,129,130,158,159,160,208,209] [41,60,61] [62] [65]
Quercetin dihydrate		*Dittrichia viscosa*	[98]
Quercetin 3,7-disulfate		*Iphiona scabra*	[96]
Quercetin 3,7,4′-trisulfate (CID: 21676176)		*Iphiona scabra*	[96]
Quercetin 3-*O*-glucoside (CID: 5280804) Synonyms: Isoquercetin, Isoquercitrin		*Anvillea garcinii* subsp. *radiata* *Asteriscus graveolens* *Blumea balsamifera*; *B. megacephala*; *B. riparia* *Buphthalmum salicifolium*; *B. speciosissimum* *Carpesium cernuum* *Chiliadenus glutinosus*; *C. montanus* *Dittrichia viscosa* *Duhaldea cappa*; *D. nervosa* *Inula anatolica*; *I. aucheriana*; *I. discoidea*; *I. helenium*; *I. inuloides*; *I. japonica*; *I. peacockiana*; *I. racemosa*; *I. sarana*; *I. sechmenii*; *I. thapsoides*; *I. viscidula* *Iphiona scabra* *Pentanema britannicum*; *P. ensifolium*; *P. mariae*; *P. oculus-christi* *Pulicaria arabica*; *P. gnaphalodes*; *P. incisa*; *P. odora*; *P. paludosa*; *P. sicula*; *P. undulata*; *P. vulgaris* *Rhanterium adpressum*	[86,142] [122] [108,123,210,211] [124,163] [125] [11,190,212] [69,99] [66,112,201] [21,40,51,53,156,174] [96] [40,213] [25,95,117,160,214,215] [41]
Quercetin 3-*O*-acetylglucoside		*Blumea megacephala*; *B. riparia*	[123]
Quercetin 3-*O*-acetylhexoside		*Inula sarana*.	[53]
Caffeoyl isoquercetin		*Blumea megacephala*; *B. riparia*	[123]
Quercetin 7-*O*-glucoside (CID: 5282160) Synonyms: Quercimeritrin; Quercimeritroside		*Anvillea garcinii* *Asteriscus graveolens* *Buphthalmum speciosissimum* *Chiliadenus glutinosus* *Inula acuminata* *Limbarda crithmoides* *Pentanema caspicum* *Pulicaria jaubertii*; *P. odora*; *P. paludosa*; *P. sicula*	[171] [122] [163] [212] [65] [216] [65] [95,164]
Quercetin 3′-*O*-glucoside (CID: 9934142)		*Pulicaria jaubertii*	[164,209]
Quercetin 4′-*O*-glucoside (CID: 5320844) Synonym: Spiraeoside		*Duhaldea cappa*; *D. cuspidata*	[65]
Quercetin 7,4′-di-*O*-glucoside (CID: 11968881)		*Inula acuminata* *Pentanema caspicum*	[65] [65]
Quercetin 3-*O*-galactoside (CID: 5281643) Synonym: Hyperoside		*Asteriscus graveolens* *Blumea balsamifera*; *B. megacephala* *Chiliadenus glutinosus* *Dittrichia graveolens*; *D. viscosa* *Inula sarana* *Iphiona scabra* *Pallenis spinosa* *Pentanema ensifolium*; *P. salicinum*; *P. spiraeifolium* *Pulicaria gnaphalodes*; *P. incisa*; *P. paludosa*; *P. schimperi*; *P. sicula*; *P. undulata*; *P. vulgaris* *Rhanterium suaveolens*	[44,122] [108,210] [11,212] [170,198] [53] [96] [149] [58,213] [95,117,118,120,121,160,168] [61,217]
Quercetin 3-*O*-(6″-caffeoylgalactopyranoside)		*Pentanema ensifolium*	[213]
Quercetin 7-*O*-galactoside (CID: 44259224)		*Inula helenium*	[174]
Quercetin 3-*O*-arabinoside (CID: 10252339) Synonym: Guaiaverin		*Blumea balsamifera* *Dittrichia viscosa*	[132] [170]
Quercetin 3-*O*-rhamnoside (CID: 5280459) Synonyms: Quercitrin; Quercitroside; Quercimelin; Thujin		*Chiliadenus montanus* *Inula japonica* *Rhanterium suaveolens*	[190] [12] [61]
Quercetin rhamnoside		*Dittrichia viscosa*	[77]
Quercetin 3-*O*-galacturonide		*Chiliadenus montanus* *Pulicaria gnaphalodes*	[190] [218]
Quercetin 3-*O*-glucuronide (CID: 5274585) Synonyms: Miquelianin; Quercituron		*Chiliadenus glutinosus*; *C. montanus* *Dittrichia viscosa* *Inula discoidea* *Pulicaria arabica*; *P. armena*; *P. dysenterica*; *P. gnaphalodes*; *P. odora*; *P. paludosa*; *P. vulgaris*	[38,169,190] [139,170] [50] [82,95,129,187,208,214,218]
Quercetin 3-*O*-glucuronide-6″-methyl ester (Artifact?)		*Chiliadenus glutinosus* *Pulicaria armena*	[169,219] [82]
Quercetin 7-*O*-glucuronide (CID: 11641481)		*Pulicaria sicula*	[95]
Quercetin *O*-hexuronide		*Telekia speciosa*	[62]
Quercetin 3-*O*-xyloside (CID: 5321278)		*Dittrichia viscosa* *Pulicaria jaubertii*	[170] [164]
Quercetin *O*-pentoside		*Rhanterium suaveolens*	[61,217]
Quercetin *O*-hexoside		*Dittrichia viscosa* *Pulicaria incisa*; *P. undulata*	[139] [130]
Quercetin *O*-hexosyl malonate		*Pulicaria incisa*	[130]
Quercetin *O*-acetylhexoside		*Pulicaria undulata*	[130]
Quercetin 3-*O*-(6″-*O*-acetyl)-hexoside		*Dittrichia viscosa*	[172]
Quercetin *O*-(caffeoyl)-hexoside		*Dittrichia viscosa*	[172]
Quercetin *O*-(*p*-coumaroyl)-hexoside		*Dittrichia viscosa*	[172]
Quercetin *O*-(feruloyl)-hexoside		*Dittrichia viscosa*	[172]
Quercetin *O*-feruloyl-*O*-hexoside		*Dittrichia viscosa*	[172]
Quercetin *O*-*p*-coumaroyl-*O*-hexoside		*Dittrichia viscosa*	[172]
Quercetin 3-*O*-rhamnoglucoside (CID: 5280805) Synonyms: Rutin; Rutoside; Phytomelin; Birutan; Quercetin 3-rutinoside; Myrticolorin		*Anvillea garcinii* *Anvillea garcinii* subsp. *radiata* *Asteriscus graveolens* *Blumea balsamifera*; *B. lacera*; *B. sinuata*; *B. megacephala*; *B. riparia* *Chiliadenus glutinosus*; *C. iphionoides* *Chrysophthalmum montanum* *Dittrichia graveolens*; *D. viscosa* *Duhaldea nervosa* *Inula acuminata*; *I. anatolica*; *I. aucheriana*; *I. discoidea*; *I. grandiflora*; *I. helenium*; *I. inuloides*; *I. japonica*; *I. montbretiana*; *I. peacockiana*; *I. racemosa*; *I. sarana*; *I. stewartii*; *I. thapsoides*; *I. viscidula* *Iphiona aucheri*; *I. grantioides* *Limbarda crithmoides* *Pallenis maritima* subsp. *maritima*; *P. spinosa* *Pentanema britannicum*; *P. caspicum*; *P. mariae*; *P. oculus-christi*; *P. orientale* *Pulicaria armena*; *P. gnaphalodes*; *P. paludosa*; *P. salviifolia*; *P. undulata* *Rhanterium suaveolens* *Vicoa divaricata*; *V. indica*	[80,220] [86] [44] [123,131,136,154,196,211,221] [75,155] [64] [65,69,99,173] [112] [40,53,65,114,115,157,203,204] [65] [205,222] [55,207,223] [40,59,65] [82,95,117,120,161,224] [60,61] [65]
Quercetin 7-*O*-rhamnoglucoside		*Inula sarana* *Pulicaria paludosa*	[53] [95]
Quercetin 3-*O*-rhamnogalactoside		*Blumea balsamifera* *Pulicaria gnaphalodes*	[136] [117]
Quercetin 3-*O*-diglucuronide		*Pulicaria paludosa*; *P. sicula*	[95]
Quercetin galactosylrhamnoside		*Dittrichia viscosa*	[69]
Quercetin 3-*O*-diglucoside 7-*O*-glucoside		*Anvillea garcinii* subsp. *radiata*	[86]
Quercetin 3-*O*-sophoroside 7-*O*-glucoside		*Inula clarkei*; *I. obtusifolia*	[65]
Quercetin 3-*O*-rutinoside 7-*O*-xyloside		*Dittrichia viscosa*	[69]
Quercetin 3-*O*-rutinoside 7-*O*-glucuronide		*Inula racemosa*; *I. stewartii*	[65]
Quercetin 3,7-di-*O*-rhamnoside		*Pulicaria undulata*	[120]
Quercetin dihexoside		*Dittrichia viscosa* *Pulicaria incisa*	[77] [130]
Quercetin 7-*O*-triglucoside		*Inula helenium*	[202]
Quercetin 3-methyl ether (CID: 5280681) Synonym: 3-*O*-Methylquercetin	5,7,3′,4′-Tetrahydroxy-3-methoxyflavone	*Allagopappus viscosissimus* *Blumea balsamifera* *Dittrichia viscosa* *Inula helenium* *Pallenis hierochuntica*; *P. spinosa* *Pentanema britannicum* *Pulicaria arabica*; *P. incisa*; *P. jaubertii*; *P. schimperi*; *P. undulata*	[175] [210,225] [47,200] [200] [54,149] [43] [118,158,159,168]
3-Methoxyquercetin 7-*O*-glucoside		*Dittrichia viscosa*	[46,200]
Rhamnetin (CID: 5281691) Synonym: Quercetin 7-methyl ether	3,5,3′,4′-Tetrahydroxy-7-methoxyflavone	*Asteriscus graveolens* *Blumea balsamifera*; *B. riparia* *Chiliadenus glutinosus* *Dittrichia viscosa* *Limbarda crithmoides* *Pulicaria dysenterica*; *P. incisa*; *P. jaubertii*; *P. undulata*	[226] [105,106,107,134,195,211,225] [38] [47,77,227] [73] [25,57,101,130,228]
Rhamnetin 3-*O*-galactoside		*Pulicaria undulata*	[67]
Rhamnetin *O*-hexoside		*Telekia speciosa*	[62]
Isorhamnetin (CID: 5281654) Synonyms: 3′-Methylquercetin; Isorhamnetol; Quercetin 3′-methyl ether; 3′-Methoxyquercetin; 3′-O-Methylquercetin; Isorhamnetine	3,5,7,4′-Tetrahydroxy-3′-methoxyflavone	*Anvillea garcinii* subsp. *radiata* *Blumea balsamifera* *Chiliadenus glutinosus* *Dittrichia viscosa* *Inula japonica*; *I. sarana* *Pentanema britannicum* *Pulicaria dysenterica*; *P. incisa*; *P. jaubertii*	[142,143,194] [132] [11,38] [47,77,110] [53,229] [97] [25,57,130,209,228]
Isorhamnetin 3-sulfate (CID: 5487766) Synonym: Persicarin		*Iphiona scabra*	[96]
Isorhamnetin 3,7-disulfate (CID: 15290611)		*Iphiona scabra*	[96]
Isorhamnetin 3,7,4′-trisulfate		*Iphiona scabra*	[96]
Isorhamnetin 3-*O*-glucoside (CID: 5318645)		*Anvillea garcinia* *Anvillea garcinii* subsp. *radiata* *Blumea balsamifera* *Buphthalmum salicifolium* *Dittrichia viscosa* *Inula sarana* *Iphiona scabra* *Pulicaria paludosa* *Telekia speciosa*	[80] [142] [132,210] [124] [99] [53] [96] [95] [62]
Isorhamnetin-3-*O*-(6″-*O*-feruloyl)-glucoside		*Dittrichia viscosa*	[98,110]
Isorhamnetin 3-*O*-galactoside (CID: 5318644) Synonym: Cacticin		*Iphiona scabra* *Pulicaria paludosa*	[96] [95]
Isorhamnetin 7-*O*-glucuronide		*Blumea megacephala*; *B. riparia*	[123]
Isorhamnetin *O*-glucuronide		*Chiliadenus glutinosus* *Dittrichia viscosa*	[75] [69]
Isorhamnetin hexoside		*Anvillea garcinii* subsp. *radiata* *Dittrichia viscosa* *Pulicaria incisa*; *P. undulata*; *P. vulgaris*	[142] [98,110,170] [72,130]
Isorhamnetin *O*-hexuronide		*Pulicaria undulata*	[130]
Isorhamnetin 7-*O*-malonylglucoside		*Blumea megacephala*; *B. riparia*	[123]
Isorhamnetin 7-*O*-protocatechuylrhamnoside		*Blumea megacephala*; *B. riparia*	[123]
Isorhamnetin 3-*O*-diglucoside		*Anvillea garcinii* subsp. *radiata*	[86,142]
Isorhamnetin 3-*O*-rhamnoglucoside (CID: 5481663) Synonyms: Narcissin; Narcissoside; Isorhamnetin 3-*O*-rutinoside		*Anvillea garcinii* *Pulicaria paludosa*	[80] [95]
Isorhamnetin *O*-rhamnoglucoside		*Chiliadenus glutinosus* *Dittrichia viscosa*	[75] [172]
Isorhamnetin 3-*O*-rhamnogalactoside		*Pulicaria paludosa*	[95]
Isorhamnetin *O*-pentosylhexoside		*Dittrichia viscosa* *Pulicaria undulata*	[172] [130]
Isorhamnetin acetyl-diglucoside		*Anvillea garcinii* subsp. *radiata*	[142]
Tamarixetin (CID: 5281699) Synonyms: 4′-Methoxyquercetin; 4′-*O*-Methylquercetin; Quercetin 4′-methyl ether	3,5,7,3′-Tetrahydroxy-4′-methoxyflavone	*Blumea balsamifera*; *B. riparia* *Inula japonica*	[105,106,181,211,230] [231]
Junsainoside A (CID: 275831051) Synonym: Tamarixetin 3-*O*-caffeoylglucoside		*Blumea megacephala*; *B. riparia*	[123]
Tamarixetin 3-*O*-robinobioside		*Asteriscus graveolens*	[122]
Quercetin 3,3′-dimethyl ether (CID: 5316900)	5,7,4′-Trihydroxy-3,3′-dimethoxyflavone	*Allagopappus canariensis* *Blumea balsamifera* *Chiliadenus iphionoides*; *C. montanus* *Dittrichia viscosa* *Pulicaria incisa*; *P. schimperi*	[232] [134,210] [145,146,176,177,189,190,192] [46,47,233] [118,234]
Quercetin 3,4′-dimethyl ether (CID: 5380905)	5,7,3′-Trihydroxy-3,4′-dimethoxyflavone	*Asteriscus graveolens* *Blumea balsamifera* *Chiliadenus montanus* *Laggera decurrens*	[122] [133] [189] [235]
Dillenetin (CID: 5487855) Synonym: Quercetin 3′,4′-dimethyl ether	3,5,7-Trihydroxy-3′,4′-dimethoxyflavone	*Blumea aromatica*; *B. balsamifera*	[104,134,210]
Ombuin (CID: 5320287) Synonyms: 7,4′-Di-*O*-methylquercetin; Quercetin 7,4′-dimethyl ether	3,5,3′-Trihydroxy-7,4′-dimethoxyflavone	*Blumea balsamifera*; *B. megacephala*; *B. riparia* *Chiliadenus montanus*	[105,108,134,195,210] [190]
Rhamnazin (CID: 5320945) Synonym: Quercetin 7,3′-dimethyl ether	3,5,4′-Trihydroxy-7,3′-dimethoxyflavone	*Perralderia coronopifolia* *Pulicaria jaubertii*	[236] [209,228]
Quercetin 3,7-dimethyl ether (CID: 5280417)	5,3′,4′-Trihydroxy-3,7-dimethoxyflavone	*Blumea aromatica*; *B. balsamifera* *Pulicaria arabica*; *P. dysenterica*; *P. incisa*; *P. schimperi*; *P. undulata*	[104,133,211] [57,118,158,160,168,179]
Quercetin 7,3′,4′-trimethyl ether (CID: 5748558)	3,7-Dihydroxy-7,3′,4′-trimethoxyflavone	*Blumea balsamifera*; *B. riparia*	[181,211,225]
Ayanin (CID: 5280682) Synonyms: 3,7,4′-Tri-*O*-methylquercetin; Quercetin 3,7,4′-trimethyl ether	5,3′-Dihydroxy-3,7,4′-trimethoxyflavone	*Blumea balsamifera* *P. canariensis*; *P. dysenterica*	[107,133] [57,237]
Pachypodol (CID: 5281677) Synonym: Quercetin 3,7,3′-trimethyl ether	5,4′-Dihydroxy-3,7,3′-trimethoxyflavone	*Blumea balsamifera* *Chiliadenus iphionoides*; *C. montanus* *Pulicaria vulgaris*	[133,136] [176,177,189] [84]
Quercetin 3,3′,4′-trimethyl ether (CID: 5383438)	5,7-Dihydroxy-3,3′,4′-trimethoxyflavone	*Allagopappus canariensis* *Blumea balsamifera* *Chiliadenus montanus* *Inula japonica* *Pulicaria canariensis*; *Pulicaria sicula*	[232] [107,134] [190,192,238] [52] [43,237]
Quercetin 3,7,3′,4′-tetramethyl ether	5-Hydroxy-3,7,3′,4′-tetramethoxyflavone	*Blumea aromatica*; *B. riparia*	[104,195]
Morin (CID: 5281670) Synonym: Aurantica	3,5,7,2′,4′-Pentahydroxyflavone	*Anvillea garcinii* subsp. *radiata*	[137]
CID: 44258717	3,5,2′-Trihydroxy-7,5′-dimethoxyflavone	*Blumea balsamifera* ^1^	[193] ^1^
6-Hydroxyquercetin (CID: 5281680) Synonyms: Quercetagetin, Quercetagenin	3,5,6,7,3′,4′-Hexahydroxyflavone	*Duhaldea cuspidata*; *D. eupatorioides* *Inula acuminata*; *I. clarkei*; *I. obtusifolia*; *I. stewartii* *Iphiona aucheri* *Pentanema britannicum*; *P. caspicum* *Pulicaria undulata* *Vicoa divaricata*	[65] [65] [65] [65] [130] [65]
Quercetagetin 7-*O*-glucoside (CID: 44259796) Synonym: Quercetagitrin		*Buphthalmum salicifolium*; *B. speciosissimum*	[124,163,239]
Quercetagetin 7-*O*-(6″-*O*-isobutyrylglucoside)		*Buphthalmum salicifolium*	[124]
Quercetagetin 7-*O*-(6″-*O*-isovalerylglucoside)		*Buphthalmum salicifolium*	[124]
Quercetagetin 7-*O*-(6″-*O*-2-methylbutyrylglucoside)		*Buphthalmum salicifolium*	[124]
Quercetagetin-*O*-hexoside		*Pulicaria undulata*	[130]
Quercetagetin-*O*-acetylhexoside		*Pulicaria undulata*	[130]
Quercetagetin-*O*-hexosylacetate		*Pulicaria undulata*	[130]
Quercetagetin 6-methyl ether (CID: 5281678) Synonyms: Patuletin; 6-*O*-Methylquercetagetin	3,5,7,3′,4′-Pentahydroxy-6-methoxyflavone	*Anvillea garcinii* subsp. *radiata* *Chiliadenus montanus* *Dittrichia viscosa* *Inula japonica*; *I. sarana* *Pallenis spinosa* *Pentanema britannicum* *Pulicaria insignis*; *P. odora* *Telekia speciosa*	[142] [189] [98] [51,53] [149] [97] [94,95] [43,62]
Patuletin glucoside		*Anvillea garcinii* subsp. *radiata*	[142]
Patuletin *O*-hexoside		*Inula sarana*	[53]
Patuletin 3-*O*-glucoside (CID: 44259782)		*Blumea lacera* *Buphthalmum salicifolium* *Chiliadenus glutinosus* *Pulicaria undulata*	[100] [124] [212] [188]
Patuletin 3-*O*-galactoside (CID: 44259776)		*Pallenis spinosa*	[149]
Patulitrin (CID: 5320435) Synonym: Patuletin 7-*O*-glucoside		*Anvillea garcinii* *Blumea lacera* *Buphthalmum salicifolium* *Chiliadenus glutinosus*; *Chiliadenus montanus* *Pallenis maritima* subsp. *maritima* *Pentanema aschersonianum*; *Pentanema britannicum* *Pulicaria odora*; *Pulicaria undulata* *Telekia speciosa*	[220] [100] [124] [190,219] [223] [56,97] [95,188] [240]
Patuletin *O*-hexoside		*Telekia speciosa*	[62]
Patuletin 7-*O*-(6″-acetyl)-glucopyranoside		*Pentanema aschersonianum*	[56]
Patuletin 7-*O*-(6″-isobutyryl)-glucoside		*Pentanema britannicum* *Buphthalmum salicifolium*	[97] [124]
Patuletin 7-*O*-[6″-(2-methylbutyryl)]glucoside		*Pentanema britannicum*	[97]
Patuletin 7-*O*-(6″-isovaleryl)glucoside		*Pentanema britannicum*	[97]
Patuletin 7-(6″-*O*-caffeoyl)glucoside		*Pallenis maritima* subsp. *maritima*	[223]
Patuletin 7-[6″(3″′-hydroxy-2″′-methylpropanoyl)] glucoside		*Pallenis maritima* subsp. *maritima*	[223,241]
Patuletin-7-[6″-*O*-caffeoyl-2″-*O*-[(S)-3′′′-hydroxy-2′′′-methylpropanoyl]glucoside] (astermaritimoside)		*Pallenis maritima* subsp. *maritima*	[223]
Patuletin 7-*O*-galactoside (CID: 44259803)		*Pallenis spinosa*	[149]
Patuletin diglucoside		*Anvillea garcinii* subsp. *radiata*	[142]
Patuletin 3-diglucoside		*Anvillea garcinii* subsp. *radiata*	[86]
Patuletin *O*-rhamnoglucoside		*Inula sarana*	[53]
Patuletin 7-diglucoside		*Anvillea garcinii* subsp. *radiata*	[86]
Patuletin 3-*O*-rharnnopyranosyl (1->6)-galactopyranoside		*Pallenis spinosa*	[148,149,207]
Quercetagetin 3′-methyl ether (CID: 10735304)	3,5,6,7,4′-Pentahydroxy-3′-methoxyflavone	*Limbarda crithmoides*	[242]
Quercetagetin 3′-methyl ether 3-*O*-rhamnoglucoside		*Blumea megacephala*; *B. riparia*	[123]
Quercetagetin 4′-methyl ether 7-*O*-caffeoylglucoside		*Blumea megacephala*; *B. riparia*	[123]
Quercetagetin methyl ether-*O*-hexoside		*Pulicaria undulata*	[130]
Quercetagetin 3,4′-dimethyl ether (CID: 5320823)	5,6,7,3′-Tetrahydroxy-3,4′-dimethoxyflavone	*Inula japonica*	[243,244]
Quercetagetin 3,7-dimethyl ether Synonym: Tomentin	5,6,3′,4′-Tetrahydroxy-3,7-dimethoxyflavone	*Pentanema britannicum*; *P. germanicum*; *P. spiraeifolium* *Pulicaria arabica*; *P. dysenterica*	[43,57] [81,187,214]
Eupatolitin (CID: 5317291) Synonym: Quercetagetin 6,7-dimethyl ether	3,5,3′,4′-Tetrahydroxy-6,7-dimethoxyflavone	*Pulicaria dysenterica*; *P. undulata*	[129,188]
Quercetagetin 6,3′-dimethyl ether (CID: 5321435) Synonyms: Spinacetin; Spinacetine; Quercetagetin 3′,6-dimethyl ether	3,5,7,4′-Tetrahydroxy-6,3′-dimethoxyflavone	*Anvillea garcinii* subsp. *radiata* *Dittrichia viscosa* *Inula japonica*; *I. sarana* *Pentanema britannicum*	[86,142] [47,77,98] [52,53,116,229] [102,245]
Spinacetin 3-*O*-glucoside (CID: 44259790)		*Asteriscus graveolens*	[226]
Spinacetin 7-*O*-glucoside (CID: 44259816)		*Anvillea garcinii* subsp. *radiata*; *A. garcinii*	[86,142,220]
Spinacetin 3-*O*-diglucoside		*Anvillea garcinii* subsp. *radiata*	[86,142]
Spinacetin 3-*O*-rhamnoglucoside		*Anvillea garcinia*; *A. graveolens*	[80,226]
Spinacetin 3-[rhamnosyl-(1->6)-glucoside] 7-rhamnopyranoside		*Anvillea garcinii*	[171]
Quercetagetin 3,6-dimethyl ether (CID: 5281603) Synonym: Axillarin	5,7,3′,4′-Tetrahydroxy-3,6-dimethoxyflavone	*Asteriscus sericeus* *Chiliadenus montanus* *Dittrichia viscosa* *Inula japonica*; *I. sarana* *Pentanema britannicum*; *P. germanicum*; *P. spiraeifolium* *Pulicaria insignis*; *P. undulata* *Telekia speciosa*	[57] [190] [47] [52,53] [43,57,97] [94,183,246] [62]
Jaceidin (CID: 5464461) Synonyms: Quercetagetin 3,6,3′-trimethyl ether; Jaceidine	5,7,4′-Trihydroxy-3,6,3′-trimethoxyflanone	*Asteriscus graveolens*; *A. sericeus* *Blumea lacera* *Chiliadenus candicans*; *C. iphionoides*; *C. montanus* *Dittrichia viscosa* *Inula sarana* *Pentanema britannicum*	[57,122] [100] [109,145,146,189,190,238] [70] [53] [43]
Centaureidin (CID: 5315773) Synonym: Quercetagetin 3,6,4′-trimethyl ether	5,7,3′-Trihydroxy-3,6,4′-trimethoxyflavone	*Blumea lacera* *Chiliadenus montanus* *Inula sarana*	[100] [189,190] [53]
Oxyayanin B (CID: 442621) Synonym: Quercetagetin 3,7,4′-trimethyl ether		*Pulicaria dysenterica*; *P. sicula*; *P. wightiana*	[43,167,247]
Quercetagetin 6,7,3′-trimethyl ether (CID: 6453535)	3,5,4′-Trihydroxy-6,7,3′-trimethoxyflavone	*Blumea lacera* *Pentanema britannicum*	[144] [102]
Eupatin (CID: 5317287) Synonym: Quercetagetin 6,7,4′-trimethyl ether; Veronicafolin	3,5,3′-Trihydroxy-6,7,4′-trimethoxyflavone	*Inula helenium*; *I. japonica* *Pulicaria dysenterica*	[52,116,147,248] [129]
Chrysosplenol C (CID: 189065) Synonyms: 6-Hydroxyquercetin 3,7,3′-trimethyl ether; Quercetagetin 3,7,3′-trimethyl ether	5,6,4′-Trihydroxy-3,7,3′-trimethoxyflavone	*Asteriscus sericeus* *Blumea balsamifera*; *B. eriantha*; *B. lacera*; *B. megacephala* *Chrysophthalmum montanum* *Duhaldea cappa* *Pentanema montanum* *Pulicaria dysenterica*; *P. inuloides*; *P. paludosa*; *P. sicula*; *P. vulgaris*	[57] [100,108,211,249] [250] [68] [92] [43,84,95,185,186,187]
Chrysosplenol D (CID: 5280699) Synonyms: Quercetagetin 3,6,7-trimethyl ether	5,3′,4′-Trihydroxy-3,6,7-trimethoxyflavone	*Blumea lacera*; *B. malcolmii* *Chiliadenus candicans*; *C. montanus* *Pentanema britannicum*; *P. germanicum*; *P. spiraeifolium* *Perralderia coronopifolia* *Pulicaria arabica*; *P. sicula*	[191,251] [189,192,252,253] [43,57] [236] [43,252]
Quercetagetin 3,5,7-trimethyl ether (CID: 14376221)	6,3′,4′-Trihydroxy-3,5,7-trimethoxyflavone	*Pulicaria arabica*	[214]
Quercetagetin 5,7,3′-trimethyl ether	3,6,4′-Trihydroxy-5,7,3′-trimethoxyflavone	*Pulicaria armena*	[82]
Quercetagetin 7,3′,4′-trimethyl ether (CID: 5322091)	3,5,6-Trihydroxy-7,3′,4′-trimethoxyflavone	*Pulicaria sicula*	[43]
Quercetagetin trimethyl ether		*Pulicaria incisa* *Telekia speciosa*	[130] [62]
Bonanzin (CID: 5379563) Synonym: Quercetagetin 3,6,3′,4′-tetramethyl ether	5,7-Dihydroxy-3,6,3′,4′-tetramethoxyflavone	*Blumea lacera* *Chiliadenus montanus*	[100] [190,192]
Chrysosplenol B (CID: 5281608) Synonyms: Chrysosplenetin; Chrysosplenetin B; Quercetagetin 3,6,7,3′-tetramethyl ether	5,4′-Dihydroxy-3,6,7,3′-tetramethoxyflavone	*Blumea lacera*; *B. malcolmii* *Chiliadenus montanus* *Duhaldea wissmanniana* *Pentanema britannicum*; *P. spiraeifolium* *Pulicaria gnaphalodes*; *P. salviifolia*; *P. sicula*	[100,191] [189,190] [113] [43,57] [43,57,119]
Casticin (CID: 5315263) Synonyms: Quercetagetin 3,6,7,4′-tetramethyl ether; Vitexicarpin	5,3′-Dihydroxy-3,6,7,4′-tetramethoxyflavone	*Chiliadenus montanus* *Inula japonica*; *I. sarana* *Pentanema spiraeifolium* *Pulicaria gnaphalodes*; *P. salviifolia*	[189] [52,53] [43] [119,218]
Quercetagetin 3,5,7,4′-tetramethyl ether (CID: 389316)	6,3′-Dihydroxy-3,5,7,4′-tetramethoxyflavone	*Chiliadenus candicans* *Pulicaria salviifolia*	[109] [254]
Quercetagetin 3,5,7,3′-tetramethyl ether (CID: 14376220)	6,4′-Dihydroxy-3,5,7,3′-tetramethoxyflavone	*Chiliadenus candicans* *Pulicaria arabica*; *P. inuloides*; *P. paludosa*; *P. sicula*	[109] [95,186,214,255]
Quercetagetin 3,7,3′,4′-tetramethyl ether (CID: 14376225)	5,6-Dihydroxy-3,7,3′,4′-tetramethoxyflavone	*Pulicaria dysenterica*; *P. inuloides*; *P. sicula*; *P. vulgaris*	[43,57,84,95,186,187]
Quercetagetin tetramethyl ether		*Pulicaria incisa*	[130]
Quercetagetin 3,6,7,3′,4′-pentamethyl ether (CID: 5320351) Synonyms: Artemetin; Artemisetin; Artemitin; Erianthin	5-Hydroxy-3,6,7,3′,4′-pentamethoxyflavone	*Blumea eriantha*; *B. lacera*; *B. malcolmii* *Chiliadenus montanus* *Duhaldea cappa*; *D. wissmanniana* *Iphiona scabra* *Pentanema britannicum*; *P. spiraeifolium* *Pulicaria sicula*	[191,251,256,257] [189,190] [68,113] [96] [43,57] [43]
Quercetagetin 3,5,6,7,3′-pentamethyl ether (CID: 14376231)	4′-Hydroxy-3,5,6,7,3′-pentamethoxyflavone	*Chiliadenus montanus* *Pallenis spinosa*	[189] [149]
Quercetagetin 3,5,7,3′,4′-pentamethyl ether	6-Hydroxy-3,5,7,3′,4′-pentamethoxyflavone	*Pulicaria arabica*	[258]
Quercetagetin pentamethyl ether		*Pulicaria odora*	[95]
Hexamethylquercetagetin (CID: 386331)	3,5,6,7,3′,4′-Hexamethoxyflavone	*Chiliadenus montanus* *Pallenis spinosa* *Pulicaria arabica*; *P. incisa*; *P. sicula*	[189] [149] [43,95,130,258]
Myricetin (CID: 5281672)	3,5,7,3′,4′,5′-Hexahydroxyflavone	*Asteriscus graveolens* *Blumea lacera*; *B. sinuata* *Dittrichia viscosa* *Inula helenium*; *I. peacockiana*	[44] [154,196,221] [135] [115,156]
Myricitrin (CID: 5281673) Synonym: Myricetin 3-rhamnoside		*Blumea balsamifera* *Carpesium nepalense*	[136] [259]
Myricetin *O*-glucuronide		*Dittrichia viscosa*	[69]
Myricetin glucoside		*Asteriscus graveolens*	[44]
Myricetin hexoside		*Dittrichia viscosa* *Pallenis spinosa*	[170] [207]
Laricitrin (CID: 5282154) Synonym: 3′-Methylmyricetin; Myricetin 3′-methyl ether	3,5,7,4′,5′-Pentahydroxy-3′-methoxyflavone		
Laricitrin 3-glucuronide		*Dittrichia viscosa*	[170]
Mearnsetin (CID: 10359384)	3,5,7,3′,5′-Pentahydroxy-4′-methoxyflavone		
Mearnsetin *O*-hexoside		*Chiliadenus glutinosus*	[38,75]
Mearnsetin *O*-glucuronide		*Chiliadenus glutinosus*	[38,75]
Mearnsetin *O*-glucuronide *O*-hexoside		*Chiliadenus glutinosus*	[75]
Trimethylmyricetin		*Dittrichia viscosa*	[135]
	3-Hydroxy-6,7,8,3′,4′-pentamethoxyflavone	*Blumea eriantha*	[260]
	3,3′,4′-Trihydroxy-6,7,8-trimethoxyflavone	*Blumea eriantha*	[260]
3-Methoxytangeretin (CID: 11741814)	3,5,6,7,8,4′-Hexamethoxyflavone	*Chiliadenus iphionoides*	[176]
CID: 13915678	5,7,2′,5′-Tetrahydroxy-3,4′-dimethoxyflavone	*Laggera decurrens*	[235]
CID: 13915679	5′-Acetoxy-5,7,2′-trihydroxy-3,4′-dimethoxyflavone	*Laggera decurrens*	[235]
Inucrithmin (CID: 10569574)	3,7,3′,4′-Tetrahydroxy-6,5′-dimethoxyflavone	*Limbarda crithmoides*	[242]
Grantiodin (CID: 44259721)	5-Hydroxy-3,6,7,2′,5′-pentamethoxyflavone	*Iphiona grantioides*	[261]
5-O-Demethylapulein (CID: 44259894)	5,2′,5′-Trihydroxy-3,6,7,4′-tetramethoxyflavone	*Duhaldea wissmanniana*	[113]
Brickellin (CID: 13871363)	5,2′-Dihydroxy-3,6,7,4′,5′-pentamethoxyflavone	*Duhaldea cappa*; *D. wissmanniana*	[68,113]
Brickellin 5-methyl ether	2′-Hydroxy-3,5,6,7,4′,5′-hexamethoxyflavone	*Pulicaria sicula*	[43]
Grantioidinin (CID: 14861189)	5-Hydroxy-3,6,7,8,2′,5′-hexamethoxyflavone	*Iphiona grantioides*	[103]

^1^ Published as a metabolite of *Inula cappa*, corrected in 1984 by Goswami et al. [262].

Flavonols oxygenated at C-8 are rare in the Inuleae-Inulinae. Flavonols with a methoxyl group at C-8 were found in *B. eriantha* [260], *C. iphionoides* [176] and *I. grantioides* [103]. Other flavonols with unique structures were japonicins A and B (Figure 2), isolated from flowers of *I. japonica* [51].

#### 2.1.3. Flavanols, Flavanones and Flavanonols of the Inuleae-Inulinae

The three subclasses of flavonoids are devoid of the C-2/C-3 double bond in the molecule. Derivatives of naringenin, eriodictyol and hesperetin (eriodictyol 4′-methyl ether) are representatives of the flavanone subclass that are often found in the Inuleae-Inulinae. Aromadendrin (dihydrokaempferol) and taxifolin (dihydroquercetin) together with its methyl ethers are the flavanonols most frequently detected in the plants of the subtribe (Table 4). Farrerol, a flavanone of an uncommon structure (6,8-dimethylflavanone) was tentatively identified in the extract from *P. undulata* [120].

**Table 4:** Flavanols, flavanones and flavanonols of the Inuleae-Inulinae.

Trivial Name of the Compound	Substitution Pattern	Plant Species	Reference
**Flavanols**			
(+)-Catechin (CID: 9064)	3,5,7,3′,4′-Pentahydroxyflavan	*Anvillea garcinii* subsp. *radiata* *Blumea balsamifera*; *B. lacera*; *B. sinuata* *Chrysophthalmum montanum* *Dittrichia graveolens*; *D. viscosa* *Duhaldea nervosa* *Inula helenium* *Pallenis spinosa* *Pulicaria incisa*; *P. undulata*	[137] [107,154,196,221] [64] [77,199] [201] [156] [55] [22,161]
Catechin hydrate		*Dittrichia viscosa*	[99]
Catechin hexoside		*Dittrichia viscosa*	[139]
Catechin gallate (CID: 6419835)		*Inula helenium*; *I. racemosa*	[21,174]
Methylcatechin		*Pulicaria vulgaris*.	[72]
(-)-Epicatechin (CID: 72276)	3,5,7,3′,4′-Pentahydroxyflavan	*Blumea lacera*; *B. sinuata* *Duhaldea nervosa* *Inula grandiflora*; *I. helenium*; *I. racemosa* *Pallenis spinosa* *Pulicaria vulgaris* *Rhanterium suaveolens*	[154,196,221] [201] [21,114,156,174] [55] [72] [61]
(+)-Gallocatechin (CID: 65084) Synonym: Gallocatechol	3,5,7,3′,4′,5′-Hexahydroxyflavan	*Chiliadenus glutinosus* *Limbarda crithmoides*	[38] [73]
Gallocatechin derivative		*Limbarda crithmoides*	[73]
(-)-Epigallocatechin (CID: 72277)		*Pallenis spinosa* *Pulicaria dysenterica*	[55] [129]
Gallocatechin/Epigallocatechin-3-gallate		*Dittrichia viscosa*	[77]
Epigallocatechin gallate		*Pentanema britannicum*	[263]
**Flavanones/Flavanonols**			
Farrerol (CID: 91144)	6,8-Dimethyl-5,7,4′-trihydroxyflavanone	*Pulicaria undulata*	[120]
Naringenin (CID: 439246) Synonym: Salipurpol	5,7,4′-Trihydroxyflavanone	*Allagopappus viscosissimus*; *A. canariensis* *Blumea balsamifera* *Chrysophthalmum montanum* *Dittrichia viscosa* *Inula anatolica*; *I. aucheriana*; *I. discoidea*; *I. helenium*; *I. inuloides*; *I. peacockiana*; *I. sarana*; *I. sechmenii*; *I. thapsoides*; *I. viscidula* *Pallenis spinosa* *Pentanema britannicum*; *P. mariae*; *P. oculus-christi* *Rhanterium suaveolens*	[175,232] [132] [64] [46,77] [39,40,53] [55] [40] [60]
Naringenin 7-*O*-hexoside		*Dittrichia viscosa*	[172]
Naringenin 7-rhamnoglucoside Synonym: Naringin		*Anvillea garcinii* subsp. *radiata* *Chiliadenus iphionoides* *Rhanterium suaveolens*	[137] [197] [61]
Naringenin 6-*C*-glucoside Synonym: Hemiphloin (CID: 160711)		*Blumea balsamifera*	[132]
Naringenin 8-*C*-glucoside Synonym: Isohemiphloin (CID: 42607891)		*Blumea balsamifera*	[225,264]
Naringenin 7-methyl ether Synonym: Sakuranetin (CID: 73571)	5,4′-Dihydroxy-7-methoxyflavanone	*Blumea balsamifera*; *B. fistulosa* *Dittrichia graveolens*; *D. viscosa* *Pulicaria incisa*	[132,150] [46,47,65,87,265] [215]
Naringenin 4′-methyl ether Synonym: Ponciretin (CID: 25201019)	5,7-Dihydroxy-4′-methoxyflavanone	*Blumea megacephala*; *B. riparia*	[123]
Ponciretin 7-*O*-glucoside (CID: 102004611) Synonym: Isosakuranin		*Pulicaria undulata*	[120]
Naringenin 7,4′-dimethyl ether (CID: 321346)	5-Hydroxy-7,4′-dimethoxyflavanone	*Carpesium lipskyi*; *C. longifolium*	[266,267]
Eriodictyol (CID: 440735) Synonyms: Eriodictiol; Huazhongilexone	5,7,3′,4′-Tetrahydroxyflavanone	*Allagopappus viscosissimus* *Blumea aromatica*; *B. balsamifera* *Dittrichia viscosa* *Pulicaria incisa*; *P. undulata*	[175] [132,138,225,230] [47] [130]
Eriodictyol 7-*O*-glucoside (CID: 134693055)		*Buphthalmum salicifolium*	[124]
Eriodictyol 3′-*O*-glucoside		*Buphthalmum salicifolium*	[124]
Eriodictyol *O*-rhamnoglucoside		*Inula sarana*	[53]
Homoeriodictyol (CID: 73635) Synonym: Eriodictyol 3′-methyl ether	5,7,4′-Trihydroxy-3′-methoxyflavanone	*Blumea aromatica*.	[104]
Hesperetin (CID: 72281) Synonyms: Eriodictyol 4′-methyl ether; Hesperitin	5,7,3′-Trihydroxy-4′-methoxyflavanone	*Dittrichia graveolens*; *D. viscosa* *Inula anatolica*; *I. aucheriana*; *I. discoidea*; *I. helenium*; *I. peacockiana*; *I. sechmenii*; *I. thapsoides*; *I. viscidula* *Pentanema britannicum*; *P. mariae*; *P. oculus-christi* *Pulicaria incisa*	[77,198,268] [39,40] [40] [130]
3-Acetoxyhesperitin		*Dittrichia viscosa*	[269]
Hesperetin 7-*O*-glucoside (CID: 147394)		*Inula stewartii*	[65]
Hesperidin (CID: 10621) Synonyms: Cirantin; Hesperidoside; Hesperetin 7-rhamnoglucoside; Hesperetin 7-rutinoside		*Chrysophthalmum montanum* *Dittrichia graveolens* *Duhaldea cuspidata*; *D. eupatorioides* *Inula acuminata*; *I. anatolica*; *I. aucheriana*; *I. discoidea*; *I. inuloides*; *I. peacockiana*; *I. racemosa*; *I. rhizocephala*; *I. sechmenii*; *I. thapsoides*; *I. viscidula* *Iphiona aucheri*; *I. grantioides* *Pentanema britannicum*; *P. capsicum*; *P. mariae*; *P. oculus-christi*	[64] [65] [65] [40,65] [65] [40,65]
Sterubin (CID: 1268276) Synonym: 7-Methyleriodictyol; Eriodictyol 7-methyl ether	5,3′,4′-Trihydroxy-7-methoxyflavanone	*Blumea balsamifera*; *B. fistulosa*; *B. riparia* *Dittrichia viscosa* *Pulicaria undulata*	[133,150,152,181,230] [47] [270]
Eriodictyol 7,3′-dimethyl ether (CID: 14235076)		*Blumea riparia*	[181]
Eriodictyol 7,4′-dimethyl ether		*Blumea riparia*	[181]
CID: 11483087	5,7,3′,5′-Tetrahydroxyflavanone	*Blumea balsamifera*	[105,106,107,211]
CID: 25073757	5,7,2′,5′-Tetrahydroxyflavanone	*Blumea balsamifera*	[133,193]
Blumeatin ^1^ (CID: 70696494)	5,3′,5′-Trihydroxy-7-methoxyflananone	*Blumea balsamifera*	[105,106,107,230,271,272]
Pinobanksin (CID: 73202)	3,5,7-Trihydroxyflavanone		
Pinobanksin 5-methyl ether 3-*O*-acetate		*Limbarda crithmoides*	[73]
Aromadendrin (CID: 122850) Synonym: Dihydrokaempferol	3,5,7,4′-Tetrahydroxyflavanone	*Carpesium macrocephalum* *Dittrichia graveolens*; *D. viscosa* *Pulicaria arabica*; *P. jaubertii*; *P. undulata*	[273] [87,172] [158,159,179]
3-*O*-Acetylaromadendrin		*Dittrichia viscosa*	[46,47,98]
Aromadendrin 7-methyl ether (CID: 181132) Synonym: 7-Methylaromadendrin	3,5,4′-Trihydroxy-7-methoxyflavanone	*Blumea balsamifera* *Dittrichia graveolens*; *D. viscosa* *Pulicaria incisa*; *P. jaubertii*; *P. undulata*	[132] [46,47,87,265] [179,234,270,274]
2*R*,3*R*-Dihydro-7-methoxykaempferol		*Dittrichia viscosa*	[233]
3-*O*-Acetyl-7-*O*-methylaromadendrin		*Dittrichia graveolens*; *D. viscosa*	[46,87]
3-*epi*-Acetyl-7-*O*-methylaromadendrin		*Dittrichia graveolens*	[87]
Aromadendrin 7,4′-dimethyl ether	3,5-Dihydroxy-7,4′-dimethoxyflavanone	*Pulicaria canariensis*	[275]
6-Methoxyaromadendrin	3,5,7,4′-tetrahydroxy-6-methoxyflavanone		
6-Methoxyaromadendrin 3-*O*-glucoside		*Pulicaria undulata*	[183]
(2*R*,3*R*)-5′-methoxy-3,5,7,2′-tetrahydroxyflavanone	3,5,7,2′-Tetrahydroxy-5′-methoxyflavanone	*Blumea balsamifera* ^2^	[193] ^2^
(2*R*,3*R*)-3,5,2′-Trihydroxy- 7,5′-dimethoxyflavanone	3,5,2′-Trihydroxy-7,5′-dimethoxyflavanone	*Blumea balsamifera*	[276]
(+)-Taxifolin (CID: 439533) Synonym: (2*R*,3*R*)-Dihydroquercetin	3,5,7,3′,4′-Pentahydroxyflavanone	*Blumea balsamifera* *Chiliadenus glutinosus* *Dittrichia viscosa* *Inula japonica* *Pentanema britannicum* *Perralderia coronopifolia* *Pulicaria arabica*; *P. jaubertii*; *P. undulata*	[132,230] [38] [69,268] [244,248] [102] [236] [158,179,209,274]
(-)-Taxifolin (CID: 712316) Synonym: (2*S*,3*S*)-Dihydroquercetin		*Pentanema britannicum*	[102]
Taxifolin hexoside		*Dittrichia viscosa*	[98,110]
Taxifolin *O*-pentoside		*Pulicaria incisa*; *P. undulata*	[130]
Taxifolin pentosyl-rutinoside		*Inula helenium*	[174]
3-*O*-Acetyltaxifolin		*Dittrichia graveolens*; *D. viscosa*	[46,47,87,265]
Taxifolin 7-methyl ether (CID: 12313900) Synonyms: Blumeatin C, Padmatin	3,5,3′,4′-Tetrahydroxy-7-methoxyflavanone	*Blumea balsamifera* *Dittrichia graveolens*; *D. viscosa* *Pulicaria incisa*; *P. jaubertii*; *P. undulata*	[132,211,225,230] [46,47,77,87] [168,179,209,274]
3-*epi*-Padmatin (CID: 11472604)		*Dittrichia graveolens*	[87]
3-*O*-Acetylpadmatin (CID: 10406203)		*Dittrichia graveolens*; *D. viscosa*	[46,47,87,265]
Taxifolin 3′-methyl ether (CID: 56658060) Synonym: Dihydroisorhamnetin	3,5,7,4′-Tetrahydroxy-3′-methoxyflavanone	*Pulicaria jaubertii*	[209,274]
Taxifolin 4′-methyl ether (2*R*,3*R*)-Dihydroquercetin 4′-methyl ether	3,5,7,3′-Tetrahydroxy-4′-methoxyflavanone	*Blumea balsamifera*; *B. fistulosa* *Pentanema britannicum* *Pulicaria undulata*	[105,106,107,133,150,211,225,230,277] [102] [101]
Taxifolin 7,3′-dimethyl ether (CID: 14353345) Synonym: Dihydroquercetin 7,3′-dimethyl ether	3,5,4′-Trihydroxy-7,3′-dimethoxyflavanone	*Blumea balsamifera* *Pulicaria jaubertii*	[152] [209,274]
Taxifolin 7,4′-dimethyl ether (2*R*,*3R*)-Dihydroquercetin 7,4′-dimethyl ether	3,5,3′-Trihydroxy-7,4′-dimethoxyflavanone	*Blumea balsamifera*; *B. fistulosa* *Pulicaria canariensis*	[105,106,107,133,150,211,230,277] [275]
Taxifolin 3,7-dimethyl ether	5,3′,4′-Trihydroxy-3,7-dimethoxyflavanone	*Pulicaria undulata*	[179]
Taxifolin 3′,4′-dimethyl ether	3,5,7-Trihydroxy-3′,4′-dimethoxyflavanone	*Pulicaria jaubertii*	[209]
Taxifolin 7,3′,4′-trimethyl ether	3,5-Dihydroxy-7,3′,4′-trimethoxyflavanone	*Pulicaria jaubertii*	[209]
2,3-Dihydroquercetagetin (CID: 25200634)	3,5,6,7,3′,4′-Hexahydroxyflavanone		
2,3-Dihydroquercetagetin 4′-methyl ether	3,5,6,7,3′-Pentahydroxy-4′-methoxyflavanone	*Inula helenium*	[147]
Ampelopsin (CID: 161557) Synonym: Dihydromyricetin	3,5,7,3′,4′,5′-Hexahydroxyflavanone		
Ampelopsin *O*-glucuronide		*Dittrichia viscosa*	[69]

^1^ In some cases, sterubin was erroneously identified as blumeatin. The structure of “putative blumeatin” was corrected by Xia et al. 2023 [278]; ^2^ published as a metabolite of *Inula cappa*, corrected in 1984 by Goswami et al. [262].

#### 2.1.4. Miscellaneous Flavonoids

**Figure 3:**
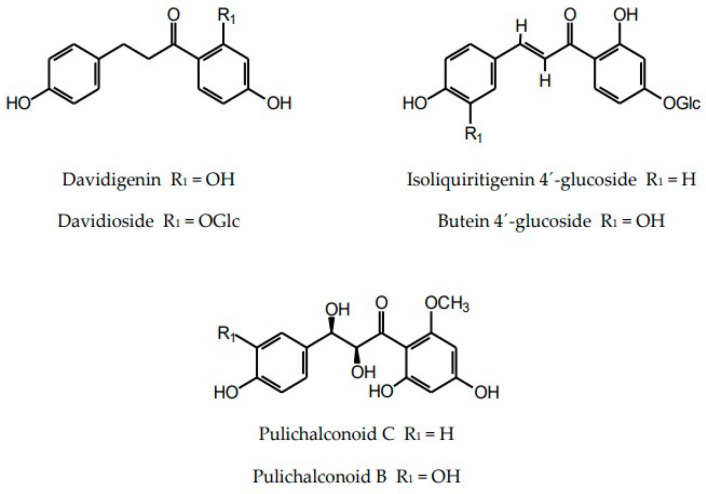
Structures of the selected chalcones from the Inuleae-Inulinae.

**Figure 4:**
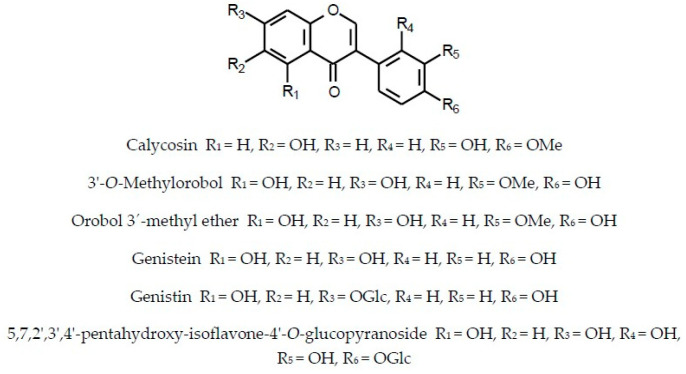
Structures of the selected isoflavones from the Inuleae-Inulinae.

Proanthocyanidins, chalcones and isoflavonoids are not common in the Inuleae-Inulinae (see Table 5). Except for davidigenin and davidioside from *B. balsamifera* [107], daidzein from *D. nervosa* [79], orobol 3′-methyl ether from *I. japonica* [51], pulichalconoids B and C from *P. incisa* [279] and 5,7,2′,3′,4′-pentahydroxyisoflavone 4′-O-glucopyranoside from *P. undulata* [160], chalcones and isoflavonoids (Figure 3 and Figure 4) were minor constituents of the analyzed plant materials and were detected in the plant extracts by TLC [65] or tentatively identified using different variants of liquid chromatography–mass spectrometry (LC-MS).

**Table 5:** Proanthocyanidins, chalcones and isoflavonoids from Inuleae-Inulinae.

Trivial Name of the Compound	Substitution Pattern	Plant Species	Reference
**Proanthocyanidins/Catechin oligomers**			
Proanthocyjanidin dimer		*Dittrichia viscosa*	[77]
Prodelphinidin B3 (CID: 13831068)		*Dittrichia viscosa*	[77]
Mahuannin G		*Inula helenium*	[280]
Chalcones			
Davidigenin (CID: 442342)	4,2′,4′-Trihydroxydihydrochalcone	*Blumea balsamifera*	[107]
Davidigenin 2′-*O*-glucoside Synonym: Davidioside (CID: 42607667)		*Blumea balsamifera*	[107]
Licuraside (CID: 14282455) Synonym: Licraside; Davidigenin 4′-apiofuranosylglucoside		*Inula helenium*; *I. racemosa*	[280]
Pulichalconoid B (CID: 102501335)	3,4,7,8,4′,6′-Hexahydroxy-2′-methoxydihydrochalcone	*Pulicaria incisa*	[279]
Pulichalconoid C (CID: 102501334)	4,7,8,4′,6′-Pentahydroxy-2′-methoxydihydrochalcone	*Pulicaria incisa*	[279]
Butein 4′-glucoside (CID: 12303942)	3,4,2′,4′-Tetrahydroxychalcone 4′-glucoside	*Inula acuminata*, *I. rhizocephala* *Pentanema caspicum*	[65] [65]
Isoliquiritigenin 4′-glucoside (CID: 5320092) Synonyms: Neoisoliquiritin; Isoneoliquiritin	4,2′,4′-Trihydroxychalcone 4′-glucoside	*Dittrichia graveolens* *Inula racemosa*; *I. royleana* *Pentanema britannicum*; *P. orientale* *Vicoa glanduligera*; *V. divaricata*; *V. indica*	[65] [65] [65] [65]
**Isoflavonoids**			
Daidzein (CID: 5281708)	7,4′-Dihydroxyisoflavone	*Duhaldea nervosa*	[79]
Genistein (CID: 5280961) Synonyms: Prunetol, Sophoricol	5,7,4′-Trihydroxyisoflavone	*Duhaldea nervosa* *Inula aucheriana*; *I. anatolica*; *I. peacockiana*; *I. sechmenii*	[112] [40]
Genistin (CID: 5281377) Synonym: Genistein 7-*O*-glucoside		*Pulicaria undulata*	[120]
6″-*O*-Malonyl genistin		*Inula helenium*; *I. racemosa*	[21,174]
Calycosin (CID: 5280448) Synonym: 3′-Hydroxyformononetin	7,3′-Dihydroxy-4′-methoxyisoflavone	*Chiliadenus glutinosus*	[38]
Orobol 3′-methyl ether (CID: 5319744)	5,7,4′-Trihydroxy-3′-methoxyisoflavone	*Chiliadenus glutinosus* *Inula japonica*	[38] [51]
5,7,2′,3′,4′-Pentahydroxyisoflavone-4′-*O*-glucopyranoside		*Pulicaria undulata*	[160]

### 2.2. Biological Activity of Flavonoids

#### 2.2.1. Biological Activity of Flavanones and Flavanonols

Sakuranetin, 7-*O*-methylaromadendrin and 3-acetyl-7-*O*-methylaromadendrin, isolated from the dried flowering aerial parts of *D. viscosa*, demonstrated in vivo anti-inflammatory activity in 2 experimental models: phospholipase A_2_ (PLA_2_)-induced mouse paw oedema (ED_50_ = 18 mg/kg and 8 mg/kg for sakuranetin and 7-*O*-methylaromadendrin, respectively) and 12-*O*-tetradecanoylphorbol 13-acetate (TPA)-induced mouse ear oedema (ED_50_ = 205 μg/ear and 185 μg/ear for sakuranetin and 3-acetyl-7-*O*-methylaromadendrin, respectively). The in vitro experiments proved that sakuranetin and 3-acetyl-7-*O*-methylaromadendrin inhibited leukotriene B_4_ production by rat peritoneal neutrophils. Moreover, sakuranetin directly inhibited the activity of 5-lipoxygenase (5-LOX). 7-*O*-Methylaromadendrin was the only compound that inhibited the secretory PLA_2_ activity. The results of in vitro experiments may explain the anti-inflammatory effects exerted by the investigated compounds [281]. 7-*O*-Methylaromadendrin from aerial parts of *D. viscosa* at a concentration of 10 μM significantly stimulated insulin-induced glucose uptake in both differentiated 3T3-L1 adipocytes and human hepatocellular liver carcinoma (HepG2) cells. Adipocytes treated with the compound demonstrated increased gene expression for the adipocyte-specific fatty acid-binding protein (aP2) and peroxisome proliferator-activated receptor γ2 (PPARγ2). The PPARγ2 protein level and lipid accumulation were also increased in the 7-*O*-methylaromadendrin-treated cells. Moreover, the compound partly recovered sensitivity to insulin in the insulin-resistant HepG2 cells. The ability to stimulate glucose uptake via PPARγ2 activation and to improve insulin resistance suggests that 7-*O*-methylaromadendrin may be a potential candidate for the management of type 2 diabetes mellitus [282]. Sakuranetin may also be useful in maintaining glucose homeostasis [283]. Marín et al. [284] proved that 7-*O*-methylaromadendrin from *D. viscosa* prevented protein carbonylation in TPA-stimulated human polymorphonuclear leukocytes. The protein carbonylation, a non-enzymatic, post-translational modification of a protein structure is associated with several pathological conditions, including arthritis and asthma. In vivo experiments on rodents demonstrated that blumeatin, isolated from *B. balsamifera*, protected the liver against pathological changes induced by carbon tetrachloride or thioacetamide intoxication [285]. The anti-inflammatory activity of the compound was confirmed in vivo by ear-swelling experiments on mice [280]. (+)-Dihydroquercetin (taxifolin) isolated from flowers of *I. japonica* demonstrated inhibitory activity against topoisomerase I (IC_50_ = 55.7 μM) and II (IC_50_ = 3.0 μM) [244]. The compound, as well as its 4′-methyl ether and 7,4′-dimethyl ether, isolated from leaves of *B. balsamifera*, turned out to be a potent inhibitor of α-glucosidase [230].

Soluble epoxide hydrolase (sEH) inhibitors are regarded as potential drug candidates to treat inflammatory and neurodegenerative diseases. Epitaxifolin, isolated from *P. britannicum*, acted as an uncompetitive inhibitor of the enzyme (IC_50_ = 6.74 μM). (2R,3R)-Dihydroquercetin and (2S,3S)-dihydroquercetin demonstrated weaker inhibitory activity towards sEH with a half-maximal inhibitory concentration of 20.54 μM and 15.57 μM, respectively [102]. Taxifolin, 7,3′-di-*O*-methyltaxifolin, 3′-*O*-methyltaxifolin and 7-*O*-methyltaxifolin from *P. jaubertii* exhibited moderate antiproliferative activity against the HCT-116 cancer cell line (IC_50_ = 32–36 μg/mL). The expression of caspase-3 and caspase-9 genes increased in the HCT-116 cells treated with the flavanonols for 48 h. The viability of the noncancerous cell line HEK-293 was much less affected [286]. Dihydroquercetin 4′-methyl ether, from *B. balsamifera* was found to overcome tumor necrosis factor (TNF)-related apoptosis-inducing ligand (TRAIL) resistance in leukemia cells [287]. The compound was also active as a tyrosinase inhibitor (IC_50_ = 115 μM; arbutin: 233 μM). In the same experiment, dihydroquercetin 7,4′-dimethyl ether demonstrated weaker activity (IC_50_ = 162 μM) [288]. Taxifolin 4′-methyl ether, isolated from *P. undulata* herb, reduced the viability of the MCF-7 human breast cancer cells in vitro. The toxicity of the compound against the noncancerous Vero (African green monkey kidney) cell line was less pronounced. In vivo, the flavanonol significantly reduced the growth of Ehrlich ascites carcinoma in mice and significantly lowered the plasma level of the vascular endothelial growth factor (VEGF) in tumor-bearing animals [101]. (2R,3S)-(-)-4′-*O*-Methyldihydroquercetin, a compound isolated from Vietnamese *B. balsamifera,* was a more potent inhibitor of xanthine oxidase than allopurinol [289]. Several flavonoids isolated from *D. viscosa* were tested for their cytotoxic and antimicrobial activities. An acylated flavanonol 3-*O*-acetylpadmatin proved to be inactive against the cell lines and microbial strains used in the assays [290].

#### 2.2.2. Biological Activity of Flavones

Hispidulin and nepetin, isolated from *D. viscosa*, markedly reduced the in vitro viability of human breast cancer (MCF-7) and human epithelial carcinoma (HEp-2) cell lines (IC_50_ = 5.87–19.50 μg/mL) whereas the growth of the Vero cell line was less affected (IC_50_ = 103.54–105.48 μg/mL). The compounds were inactive against *Candida albicans* and four strains of bacteria (including methicillin-resistant *Staphylococcus aureus* and *Escherichia coli*) [290]. Except for the Inuleae-Inulinae, hispidulin has been isolated from several different plant species (*Centaurea* spp., *Onopordum* spp. and others). Studies on the anticancer activity of the flavone in vitro against human cancer cell lines and in vivo in different animal models have been recently summarized by Ashaq and coworkers [291]. SARS-CoV-2 3C-like protease (3CLpro) has been regarded as a target enzyme for suppressing the proliferation of SARS-CoV-2. In a search for the antiviral compounds, a series of flavonoids isolated from flowers of *P. britannicum* was investigated for the potential 3CLpro inhibitory activity. Hispidulin and nepetin were found to be competitive inhibitors of the enzyme with IC_50_ = 42.0 μM and 31.7 μM, respectively [292]. Hispidulin, luteolin, nepetin, nepitrin, hispiduloside and jaceoside, isolated from flowers of *P. montanum*, inhibited NO production (IC_50_ = 0.34–3.04 μM) in murine macrophages (RAW 267.4) stimulated with lipopolysaccharide (LPS). The compounds, in the described assay, were more active than dexamethasone (IC_50_ = 3.89 μM) [92]. According to Başpınar and coworkers [82], luteolin and a mixture of 6-hydroxyapigenin 7-methyl ether and 6-hydroxyluteolin 7,4′-dimethyl ether, isolated from *P. armena*, were not active against *Pseudomonas aeruginosa*, *S. aureus* and *C. albicans* at concentrations up to 200 μg/mL. The compounds demonstrated moderate, nonselective cytotoxic activity against human cancer cells (lines A549 and HCT116) in vitro. Moreover, luteolin showed moderate anti-quorum sensing activity against biosensor strains *Chromobacterium violaceum* CV026 and *Serratia marcescens* ATCC 27117.

Nepetin is one of the major flavonoid constituents of *Flos Inuleae*, a remedy used in commercial traditional Chinese medicine (TCM) [229]. Pretreatment or post-treatment with nepetin (1–50 μM) protected rat cortical cells against glutamate-induced damage. The protection was also effective against toxicity induced by N-methyl-D-aspartate (NMDA) and kainic acid [293]. The flavone may have a therapeutic effect in mast cell-mediated inflammatory diseases. Nepetin, at concentrations of 1.6 and 3.1 μM, significantly reduced the generation of leukotriene C_4_ (LTC_4_) and prostaglandin D_2_ (PGD_2_) by the mouse bone marrow-derived mast cells stimulated with IgE/antigen in vitro. The antiallergic activity was confirmed in vivo using a passive cutaneous anaphylaxis (PCA) reaction model in mice [294]. Nepetin from *P. insignis* demonstrated cytotoxic activity towards HeLa and HepG2 human cancer cell lines (IC_50_: 3.61–3.98 μM) but was inactive against MGC803 and T24 human cancer cells [94].

Luteolin, the most frequently found flavone constituent of Inuleae-Inulinae and a ubiquitous dietary flavonoid has been studied for its biological activity in different in vitro and in vivo experimental models [295,296,297,298]. The compound, isolated from leaves of *B. balsamifera*, inhibited xanthine oxidase with IC_50_ = 2.38 μM (IC_50_ for allopurinol: 0.97 μM) [299] and was one of the most effective sEH inhibitors derived from *P. britannicum* flowers [102]. Due to the sEH inhibitory activity, luteolin protected lungs against particulate matter 2.5 (PM 2.5)-mediated injury in mice [300]. Luteolin, from flowers of *I. japonica,* demonstrated inhibitory activity towards topoisomerase I (IC_50_ = 37 μM; camptothecin: 24.5 μM) and topoisomerase II (IC_50_ = 9.9 μM; etoposide: 26.9 μM) [244]. Moreover, the flavone dose-dependently, starting from a concentration of 10 μM, inhibited differentiation of 3T3-L1 cells into adipocytes and enhanced differentiation of the mouse myoblast cells (C2C12) that may lead to obesity alleviation and enhancement of endurance [18]. 

Luteolin 3′-methyl ether (chrysoeriol) from *P. britannicum*, based on in silico studies, was selected as a potential inhibitor of dihydrofolate reductase (DHFR-1) and may be considered as a potential therapeutic agent in *Shigella dysenteriae* type 1 infections [301].

A C-8 methoxylated flavone from *C. iphionoides*, xanthomicrol, demonstrated antifungal activity [145] and inhibited aggregation of human blood platelets induced by collagen and ADP [146].

#### 2.2.3. Biological Activity of Flavonols

Flavonols, ubiquitous constituents of plants and plant foods, have been extensively investigated with respect to their potential risks and benefits to human health. The best-known plant metabolites of this type are kaempferol, quercetin and their corresponding 3-*O*-glucosides: astragalin and isoquercetin. The pharmacological activities of flavonols and their role as components of the human diet were discussed in several review papers [302,303,304,305,306,307].

Antioxidant and α-glucosidase inhibitory activities of seven flavonol 3-methyl ethers from aerial parts of *C. iphionoides* were assayed in vitro by Al-Dabbas and coworkers [176]. Quercetin 3,3′-dimethyl ether and 6-methoxykaempferol 3-methyl ether were proved to be the best antioxidants among the investigated compounds, whereas kaempferol 3-methyl ether demonstrated the best α-glucosidase inhibitory activity. 6-Methoxykaempferol 3-methyl ether and quercetin 3,3′-dimethyl ether exerted moderate cytotoxic effects on human leukemia (HL-60) cells [177]. 6-Methoxykaempferol and quercetagetin 6,7-dimethyl ether, from aerial parts of *P. undulata*, significantly reduced the viability of MCF-7 and Hep G2 cancer cells (IC_50_: 23.5–40.2 μg/mL). The 2,2-Diphenyl-1-picrylhydrazyl (DPPH) radical scavenging activity of 6-methoxykaempferol was comparable to that of vitamin C [188]. 6-Hydroxykaempferol 3,7-dimethyl ether and chrysosplenol C from *P. inuloides* were moderately (IC_50_: 16.8–19.6 μg/mL) but selectively active against prostate cancer cell lines (PC3) resistant to doxorubicin [186]. 6-Methoxykaempferol 3,4′-dimethyl ether (santin), isolated from the inflorescences of *P. insignis*, exerted a cytotoxic effect on MGC-803, HeLa, Hep G2, and T24 human cancer cell lines (IC_50_: 3.71–4.78 μM) whereas the corresponding IC_50_ values for 6-methoxykaempferol 3-methyl ether and 6-methoxyquercetin 3-methyl ether, isolated from the same plant material, were higher than 40 μM [94].

Quercetin and tamarixetin (quercetin 4′-methyl ether), from leaves of *B. balsamifera*, showed inhibitory activity against xanthine oxidase (IC_50_: 2.92–3.16 μM; allopurinol: 0.97 μM) [299]. The activity was confirmed in the assay conducted using quercetin and quercetin 3,3′,4′-trimethyl ether isolated from *B. balsamifera* plants of Vietnamese origin (IC_50_: 1.28–1.91 μM; allopurinol: 2.50 μM) [289]. Quercetin and rhamnetin (quercetin 7-methyl ether) were competitive inhibitors of mushroom tyrosinase (IC_50_: 96–107 μM; arbutin: 233 μM) [288] and tamarixetin was a somewhat weaker inhibitor of the enzyme (IC_50_ = 144 μM) than the flavonols mentioned above.

Two quercetin glycosides, isoquercetin and quercimeritrin, isolated from *P. jaubertii*, suppressed mutant K-Ras/B-Raf protein expression and interaction in both human lung cancer (A549) and hepatocellular carcinoma (HepG2) cells. The compounds repressed IL-8 and TGF-β signaling in the treated cells, which may suggest their potential regulatory influence on the angiogenesis and metastatic ability of cancer cells [164]. Quercetin 3-methyl ether and quercetin 3,3′-dimethyl ether from *D. viscosa* demonstrated antiproliferative activity towards MCF-7 cells (IC_50_: 11.23 and 10.11 μg/mL, respectively; vincristine sulfate IC_50_ = 10.03 μg/mL) and HEp-2 cells (IC_50_: 26.12 and 28.01 μg/mL, respectively). The half-maximal inhibitory concentrations of the compounds against Vero cells were higher than 150 μg/mL. Moreover, the compounds showed moderate antibacterial activity (MIC: 62.5–125 μg/mL) against *Bacillus cereus* and *Salmonella typhimurium* [290]. Quercetin 7-methyl ether from *P. undulata* reduced the viability of MCF-7 cells in vitro (IC_50_: 18.50 μg/mL). The compound was less cytotoxic against Vero cells (IC_50_ > 50 μg/mL). In vivo, the flavonol significantly inhibited the growth of Ehrlich ascites carcinoma in mice and normalized the VEGF levels in the serum of the tumor-bearing animals [101]. An expression of the hematopoietic progenitor cell antigen CD34, a marker of angiogenesis, was also significantly reduced in the tumor tissue of the rhamnetin-treated mice [101].

The bio-guided fractionation of *L. crithmoides* flower extract led to the isolation of quercetin and quercimeritrin as constituents responsible for the antioxidative activity of the plant material [216]. Quercetin and its 3-*O*-galactoside from *P. undulata* demonstrated high DPPH radical scavenging activity (IC_50_: 7.5 and 11.4 μM, respectively). Quercetin and its 3,7-dimethyl ether, isolated from the same source, protected the hepatoma Hepa1c1c7 cell line against tert-butyl hydroperoxide (TBHP)-induced damage (EC_50_ for quercetin 3,7-dimethyl ether: 33.6 μM) [160]. Moreover, quercetin 3,7-dimethyl ether extracted from leaves of *B. balsamifera* inhibited plasmin activity (IC_50_: 1.5 μM) [133]. Tamarixetin from *B. balsamifera* turned out to be a potent DPPH scavenger (IC_50_ = 0.88 μg/mL) and inhibitor of α-glucosidase (IC_50_ = 28.0 μg/mL; acarbose 261.5 μg/mL). Quercetin 3,3′-dimethyl ether, isolated from the same plant material, had significantly lower radical scavenging activity and was less efficient in inhibiting α-glucosidase (IC_50_ = 76.85 μg/mL) than tamarixetin [230]. Pan and coworkers studied the effect of tamarixetin, isolated from flowers of *I. japonica*, on the production of inflammatory mediators by IgE/antigen-induced mouse bone marrow-derived mast cells. Flavonol decreased degranulation and the eicosanoid (LTC_4_ and PGD_2_) generation in the cells which may be useful in the prevention of allergic inflammatory diseases [231]. Ayanin (quercetin 3,7,4′-trimethyl ether), a constituent of *B. balsamifera*, *D. viscosa* and several other anti-inflammatory plant extracts, based on a virtual screening, was predicted to act as an inhibitor of human inhibitor NF-κB kinase 2 (hIKK-2) [308]. Another trimethyl ether of quercetin, pachypodol, at a concentration range of 1–5 μg/mL, completely suppressed replication of poliovirus type 1 in HeLa cells [309].

Quercetin, quercetin 3-*O*-glucoside, patuletin 3-*O*-glucoside and quercetagetin 7-*O*-glucoside (the latter compound isolated from flowers of *B. salicifolium*) scavenged reactive oxygen species (ROS) generated by the polymorphonuclear leukocytes stimulated by N-formyl-methionyl-leucyl-phenylalanine (FMLP) (72.3–81.4% inhibition at a concentration of 1 μM) or opsonized zymosan (18.1–24.7% inhibition; 1 μM) [239]. Patuletin and axillarin from flowers of *P. britannicum*, in a dose-dependent mode, protected in vitro cultured rat cortical neurons against glutamate-induced injury, when applied 1 h before or 30 min after the glutamate insult. The flavonols also provided an effective protection of the cells against both N-methyl-D-aspartate (NMDA) and kainic acid-induced neuronal damage [293]. Patuletin at a dose of 30 mg/kg (i.p.) demonstrated significant antinociceptive activity in mice, in several pharmacological tests (tail-flick test, writhing test, formalin-induced paw licking and glutamate-induced paw licking) [310]. Its mechanism of action, however, has remained unclear. Like the flavones nepetin and hispidulin, patuletin turned out to be a competitive inhibitor of SARS-CoV-2 3CLpro [292].

Quercetagetin 3,4′-dimethyl ether, obtained from the flowers of *I. japonica*, inhibited (conc. 2.9 and 29 μM) adriamycin-induced senescence and replicative senescence in human umbilical vein endothelial cells (HUVECs) in vitro [245]. The compound suppressed intracellular ROS generation triggered by adriamycin [245], inhibited topoisomerase II (IC_50_ = 6.9 μM) and was moderately cytotoxic against human lung carcinoma (A459; IC_50_ = 59.3 μM) and human colon adenocarcinoma (HT-29; IC_50_ = 30.9 μM) cell lines in vitro [244]. Another flavonol of the same origin, spinacetin, at a concentration range of 1–5 μM, significantly suppressed histamine release, Ca^2+^ mobilization, LTC_4_ generation, cPLA_2_ translocation and MAPKs phosphorylation and decreased IL-6 and COX-2 expression in bone marrow-derived mast cells activated by IgE/antigen. Peroral administration of spinacetin (25 and 50 mg/kg), dose-dependently attenuated an IgE/Ag-mediated passive cutaneous anaphylactic reaction in a mouse model [311]. Spinacetin and 3,5,4′-trihydroxy-6,7,3′-trimethoxyflavone demonstrated sEH inhibitory activity in vitro (IC_50_: 16.58 μM and 14.13 μM, respectively) that supported their role as anti-inflammatory agents [102]. Quercetagetin 3,7,3′-trimethyl ether (chrysosplenol C) from *P. armena* and *P. inuloides* demonstrated moderate cytotoxicity towards A549, HCT116 and PC3 human cancer cell lines in vitro (IC_50_: 16.8–20.0 μg/mL) [82,186]. According to Ayaz et al. [250], chrysosplenol C extracted from *C. montanum*, was cytotoxic against the human breast (MCF-7), cervical (HeLa) and lung (A549) cancer cell lines (conc. 20 μg/mL), but the activity was not clinically significant and not selective. The same compound, isolated from other plant sources, showed antiviral activity [312] and a positive inotropic effect in rat cardiac myocytes [313]. Elhady and coworkers investigated the antitumor activity of jaceidin (quercetagetin 3,6,3′-trimethyl ether) from aerial parts of *C. montanus* both in vitro and in vivo [238]. The flavonol was cytotoxic against the MCF-7 and HepG2 cancer cells (IC_50_: 9.3 and 9.7 μM, respectively) and seemed to be devoid of toxicity towards the normal human melanocytes (HFB-4) in vitro. In vivo, the compound was tested against Ehrlich’s ascites carcinoma solid tumors grown in female mice. At a dose of 50 mg/kg, jaceidin significantly reduced the tumor weight, the number of giant cells in the tumor tissue and lowered the serum level of VEGF-B. The compound, extracted from aerial parts of *C. iphionoides*, demonstrated significant antioxidant and radical scavenging activities, increased blood clotting time and exerted a thrombolytic effect in vitro [314]. Other flavonols from *C. iphionoides*, kaempferol 3,7-dimethyl ether (kumatakenin) and quercetin 3,3′-dimethyl ether demonstrated antifungal activity and inhibited aggregation of human blood platelets induced by both ADP and collagen [145,146]. Quercetagetin 6,7,4′-trimethyl ether, based on in silico studies on *P. britannicum* metabolites, was selected for further investigation as a potential inhibitor of dihydrofolate reductase that may find use in the therapy of shigellosis [301].

Bio-guided fractionation of a chloroform extract from aerial parts of *P. inuloides* led to the isolation of quercetagetin 3,5,7,3′-tetramethyl ether as a compound responsible for the leishmanicidal activity of the plant material [255]. Quercetagetin 3,5,7,4′-tetramethyl ether from aerial parts of *P. salviifolia*, at a dose of 50 mg/kg, lowered cholesterol levels (by c. 20%) in the blood serum of both healthy and hyperlipidemic rats [254].

#### 2.2.4. Biological Activity of Chalcones

Data on the activity of chalcones isolated from the Inuleae-Inulinae are sparse. Only four compounds of this structural type: davidigenin, davidioside and pulichalconoides B and C have been isolated from the plants of the tribe [107,279]. Butein 4′-O-glucoside and isoliquiritigenin 4′-O-glucoside were identified in the plant extracts by TLC [65] and their presence in the analyzed plant materials needs confirmation by other analytical methods. Licuraside was tentatively identified in the extract from ‘tumuxiang’, a traditional Chinese medicine (TCM) preparation composed of *I. helenium* and *I. racemosa* dried roots [280]. Davidigenin and davidioside isolated from *B. balsamifera* [107] were not assayed for their biological activities, but the activity of davidigenin as an aldose reductase inhibitor, inhibitor of leukotriene release from the stimulated human polymorphonuclear leukocytes and antispasmodic agent has been reported in the literature [315,316,317].

Pulichalconoid B from *P. incisa* protected rat primary astrocytes against H_2_O_2_ cytotoxicity and inhibited H_2_O_2_-induced intracellular ROS production. Treatment with pulichalconoid B increased the level of glial-derived neurotrophic factor (GDNF) transcript in the cells [279]. Moreover, pulichalconoid B at a concentration of 63 μM and 125 μM significantly inhibited the secretion of cytokines (IL-2, IL-6, IL-10, IL-12, and IFN-γ) from the LPS-stimulated mouse splenocytes [318]. In the oxazolone model of cutaneous dermatitis in mice, pulichalconoid B (at a dose of 10 mg/kg) downregulated levels of the cytokines in the supernatants of ear homogenates from oxazolone-treated mice and reduced oxazolone-induced ear edema [318].

#### 2.2.5. Biological Activity of Isoflavones

A majority of the isoflavonoids described as metabolites of Inulae-Inulinae was tentatively identified in the plant material using different variants of the HPLC-MS technique [21,38,40,51,112,120,174]. Daidzein was one of the compounds isolated from the aerial parts of *D. nervosa* of Chinese origin [79]. The isoflavone was assayed for anti-inflammatory activity in vitro by the measurement of the secretion of inflammatory cytokines (TNF-α, IL-6 and IL-1β) in the LPS-stimulated RAW 264.7 cells, pretreated with the compound, but was judged as inactive based on its IC_50_ values [79]. 5,7,2′,3′,4′-Pentahydroxyisoflavone 4′-*O*-glucoside extracted from the whole plant of *P. undulata*, turned out to be an excellent DPPH radical scavenger in vitro (IC_50_ = 3.9 μM; quercetin: 7.5 μM) but failed to protect Hepa1c1c7 murine hepatoma cells from the *tert*-butyl peroxide-induced oxidative damage [160].

### 2.3. Hydroxycinnamates

This group of phenolic compounds comprises numerous conjugates of hydroxycinnamic (caffeic, ferulic and *p*-coumaric) acids with quinic, shikimic, tartaric and aldaric acids. The most frequently isolated and identified hydroxycinnamates of the Inuleae-Inulinae are chlorogenic acids (CGAs), i.e., esters of *trans* hydroxycinnamic acids with 1L-(-)-quinic acid. The compounds were found in nearly all the examined species from the subtribe. One of the most common caffeoylquinic acids, chlorogenic acid, according to the current IUPAC rules denoted as 5-*O*-caffeoylquinic acid (5-CQA), was formerly known as 3-*O*-caffeoylquinic acid (3-CQA). In this review, the current IUPAC numbering rules have been applied but the numbering system used by the authors of papers cited herein was not always clear.

CGAs are ubiquitous plant metabolites and common constituents of food. They are present in coffee, potatoes, apples, artichokes, plums, cherries, prunes, tomatoes and carrots [319,320]. The questions concerning the chemistry, bioavailability and pharmacological activity of CGAs have been recently summarized by Clifford et al. [320] and Magaña et al. [321]. CGAs act as the antioxidative and anti-inflammatory agents that demonstrate neuroprotective effects, prevent hypoxia-induced retinal degeneration and counteract the formation of advanced glycation end products [320,322,323,324,325].

Danino and coworkers [326] proved in a series of experiments that 1,3-dicaffeoylquinic acid (1,3-DCQA), isolated from *D. viscosa*, is a potent antioxidant and may inhibit ROS generation in the growing cells. However, the IC_50_ values of 1,3-DCQA in different experimental models were lower when compared to those of the standard antioxidant compounds (caffeic acid, ferulic acid, ascorbic acid, Trolox). Fractionation of the root extract from *L. crithmoides* subsp. *crithmoides*, directed by the hepatoprotective activity, led to the isolation of 3,5-DCQA 1-methyl ether, 4,5-DCQA 1 methyl ether and 1,5-DCQA as the active metabolites of the plant [327]. Jallali et al. [216], in a search for potent antioxidants, conducted a bio-guided fractionation of the extract from flowers of *L. crithmoides.* As a result, 5-CQA, 1,5-DCQA and 3-*p*-coumaroyl-5-caffeoylquinic acid were isolated and identified, in addition to quercetin and quercimeritrin.

Methanol extract from the leaves of *D. viscosa* (40 mg/kg/day) counteracted hypertension induced by the N-Nitro-L-arginine methylester (L-NMAE) treatment in rats. A similar effect was achieved with enalapril (15 mg/kg/day). Fractions of the extract that demonstrated the best vasorelaxant effect contained 5-CQA and cynarine (1,3-DCQA). The vasorelaxant activity of the hydroxycinnamates was confirmed using the commercial standards of cynarin and chlorogenic acid [20].

5-CQA, 3,5-DCQA and 1,5-DCQA isolated from flowers of *P. montanum* inhibited NO release by the murine macrophages (RAW 267.4) stimulated with LPS (IC_50_: 31.5, 6.9 and 2.5 μM, respectively), which indicates the anti-inflammatory activity of the compounds [92]. The enzyme soluble epoxide hydrolase (sEH) has attracted some attention as a potential target for the treatment of inflammatory diseases. Zhao et al. [102] investigated the sEH inhibitory activity of 3,5-DCQA and 1,5-DCQA (although the figure shown in the paper suggested 1,3-DCQA according to IUPAC rules) in a cell-free experimental model. They proved that the studied hydroxycinnamates inhibited the target enzyme (IC_50_: 17.2 μM and 10.7 μM, respectively) as its uncompetitive inhibitors.

Murlanova and coworkers studied the antidepressant-like activity of the root extract from *D. viscosa* [27]. A fractionation of the crude extract led to the identification of fractions active against H_2_O_2_-induced damage in the rat pheochromocytoma (PC12) cells. The fraction that demonstrated the best cytoprotective effect, injected peritoneally (5–25 mg/kg), reduced immobility time in the forced swim test on mice and produced antidepressant-like effects similar to paroxetine (10 mg/kg). Moreover, the treatment with the active fraction caused neurochemical alterations comparable to the effects of paroxetine. Two major components of the active fraction from the root extract, which represented approximately 87% of the total content, were tentatively identified as 5-CQA (49%) and 1,3-DCQA (38%).

Except for the most frequently found caffeoylquinic acids, monoacyl-, diacyl- and triacylquinic acids (conjugates with ferulic, *p*-coumaric and caffeic acid) or caffeoylquinates substituted with short-chain organic acids (isobutyric, methylbutyric and others) were often identified in the extracts of Inuleae-Inulinae, mostly using HPLC-MS techniques [53,62,75,98,123,124,130,142,163,170,174,186,217,226,328,329,330,331,332]. Metabolomic studies, performed using the hyphenated methods, revealed the presence of conjugates of hydroxycinnamic acids with shikimic acid in *D. cappa* [329], *D. nervosa* [330] and *D. viscosa* [172] and conjugates of hydroxycinnamic acids with aldaric acids in *B. megacephala*, *B. riparia* [123], *B. speciosissimum* [163], *C. divaricatum* [328], *C. glutinosus* [75], *D. viscosa* [135,333], *I. helenium* [334], *I. japonica* [71], *I. sarana* [53], *P. spinosa* [207], *P. vulgaris* [72] and *P. inuloides* [186]. Caffeoyltartaric acid (caftaric acid) and 2,3-dicaffeoyltartaric acid (chicoric acid) were isolated from the roots of *I. helenium* [335]. The latter compound was also identified in the extract from *C. montanum* [64]. Another biologically active antioxidant from the hydroxycinnamate group, rosmarinic acid, was identified in *B. lacera* [154], *B. sinuata* [196], *C. montanum* [64], *D. viscosa* [77,268] and *R. suaveolens* [61]. Salvianolic acid A was tentatively identified in the extract from leaves of *D. viscosa* [173]. An analysis of the extract from *I. helenium* roots revealed the presence of galloyl-caffeoyl-hexose [174]. Caffeoyl-N-tryptophan-rhamnoside and caffeoyl-N-tryptophan were identified in the extracts from *D. viscosa* [69,172]. 

### 2.4. Flavonolignans

Except for silybin and isosilybin isolated as a mixture from *C. faberi* [63], anthelminthicol A from *P. caspicum* [336] and flavalignans cinchonain I and II, tentatively identified in the extract from leaves of *D. viscosa*, the occurrence of flavonolignans in the Inuleae-Inulinae seems to be limited to *Duhaldea* spp. [66,113,337,338]. (-)-Hydnocarpin-7-*O*-glucoside [337] and hydnocarpin D [66] were isolated from *D. cappa*. The compounds were not assayed for their biological activity but the protective role of hydnocarpin D in LPS-induced acute lung injury has been recently studied by Hong and coworkers [339]. The compound was also found to be active as a ferroptosis inducer in T-cell acute lymphoblastic leukemia cells [340].

**Figure 5:**
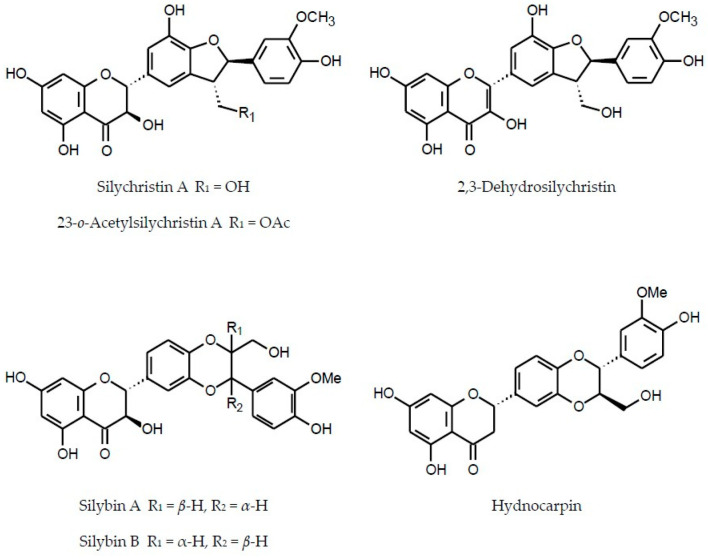
Structures of the selected flavonolignans from the Inuleae-Inulinae.

Aerial parts of *D. wissmanniana* yielded 23-*O*-acetylsilychristin A, silychristin A, silychristin B, isosilychristin, isohydnocarpin, 2,3-dehydrosilychristin, silybin A, silybin B, isosilybin A, hydnocarpin and silydianin (see Figure 5) [113,338]. Silychristin A (CID: 441764) dominated the fraction of flavonolignans [338]. Anti-inflammatory activities of the isolated compounds were assessed by the measurement of the nitrite concentration in the culture supernatant from RAW 264.7 macrophage pretreated with the flavonolignans and stimulated with LPS. 2,3-Dehydrosilychristin and hydnocarpin demonstrated moderate anti-inflammatory effects (IC_50_: 19.6 μM and 23.3 μM, respectively) under the experimental conditions of the study. Chemistry, bioavailability and pharmacological activity of silymarin, a mixture of flavonolignans extracted from *Silybum marianum* (L.) Gaertn. (Asteraceae, Cardueae) containing silybin, isosilybin, silychristin, silydianin and 2,3-dehydrosilybin as major constituents, has been discussed by Křen and Valentová in their recent review [341].

### 2.5. Lignans

**Figure 6:**
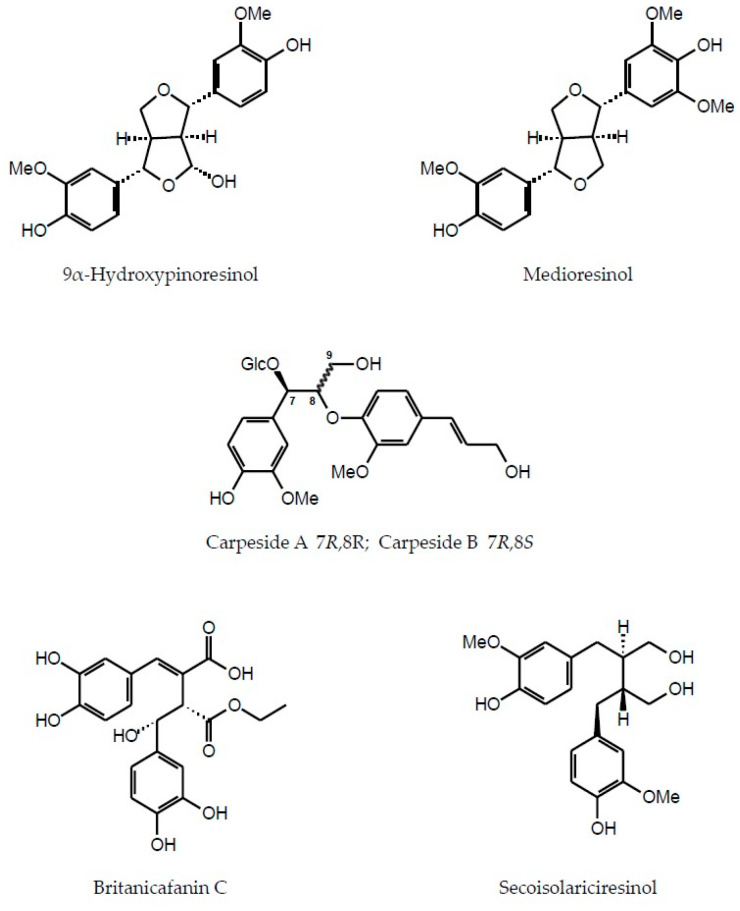
Structures of the selected Inuleae-Inulinae lignans.

This group of polyphenols comprises monolignol (*p*-coumaroyl alcohol, coniferyl alcohol and sinapyl alcohol) dimers of diverse structures (Figure 6) and different biological activity profiles [342,343,344]. Furofuran-type lignans (pinoresinol, syringaresinol and medioresinol) were the most frequently isolated lignan constituents of the Inuleae-Inulinae. They were found in the genera *Rhanterium* (*R. suaveolens*) [345], *Pulicaria* (*P. insignis*) [94], *Inula* (*I. helenium*, *I. hookeri*, *I. japonica*) [147,178,244], *Chrysophthalmum* (*C. montanum*) [250] and *Carpesium* (*C. cernuum*, *C. faberi*) [63,125]. Moreover, their presence was tentatively confirmed in *D. viscosa* and *C. glutinosus* by HPLC-MS analyses [38,75,77]. Syringaresinol from the flowers of *I. japonica* inhibited topoisomerase II (IC_50_ = 28.9 μM) and demonstrated moderate cytotoxic activity against HepG2 and HT-29 cancer cell lines (IC_50_: 30.0 μM and 57.5 μM, respectively) [244].

Neoolivil 9′-*O*-glucoside from *C. cernuum* [125] and rhanteriol [345] are representatives of tetrahydrofuranoid-type lignans. Rhanteriol has been recently isolated from aerial parts of *R. suaveolens*. The compound inhibited α-amylase (IC_50_: 46.42 μM; reference acarbose IC_50_: 5.65 μM) and α-glucosidase (IC_50_: 26.76 μM; acarbose IC_50_: 241.32 μM), as well as butyrylcholinesterase (IC_50_: 10.41 μM; reference galanthamine IC_50_: 11.63 μM), which may suggest its potential usefulness to prevent type 2 diabetes mellitus and dementia. Its acetylcholinesterase (AChE) inhibitory activity, however, was surprisingly low (21% of inhibition at 100 μM).

Britanicafanins C-E, dibenzylbutane lignans were isolated from *I. britannica* in a search of the in vitro active sEH inhibitors. In the applied assay, britanicafanins C and E were moderately active against the enzyme (IC_50_: 26.67 μM and 20.66 μM, respectively), whereas structurally closely related britanicafanin D and ternifoliuslignan A (an aryltetralin-type lignan) turned out to be inactive [102]. Another dibenzylbutane lignan, secoisolariciresinol, was tentatively identified in the extract from *Iphiona mucronata* by Pecio et al. [76].

Ceplignan, a neolignan of dihydrobenzofuran type, was isolated from *D. cappa* by Wu et al. [49], and two other neolignans of the same structural type, derivatives of blechnic acid, were tentatively identified in the extract from leaves of *D. viscosa* [172]. Citrusin A and two more 8,4′-oxyneolignans, carpesides A and B, were obtained by Ma and coworkers from the aerial parts of *C. cernuum* [125]. A biphenyl neolignan, honokiol, was tentatively identified in the extract from *C. glutinosus* [38].

### 2.6. Coumarins

**Figure 7:**
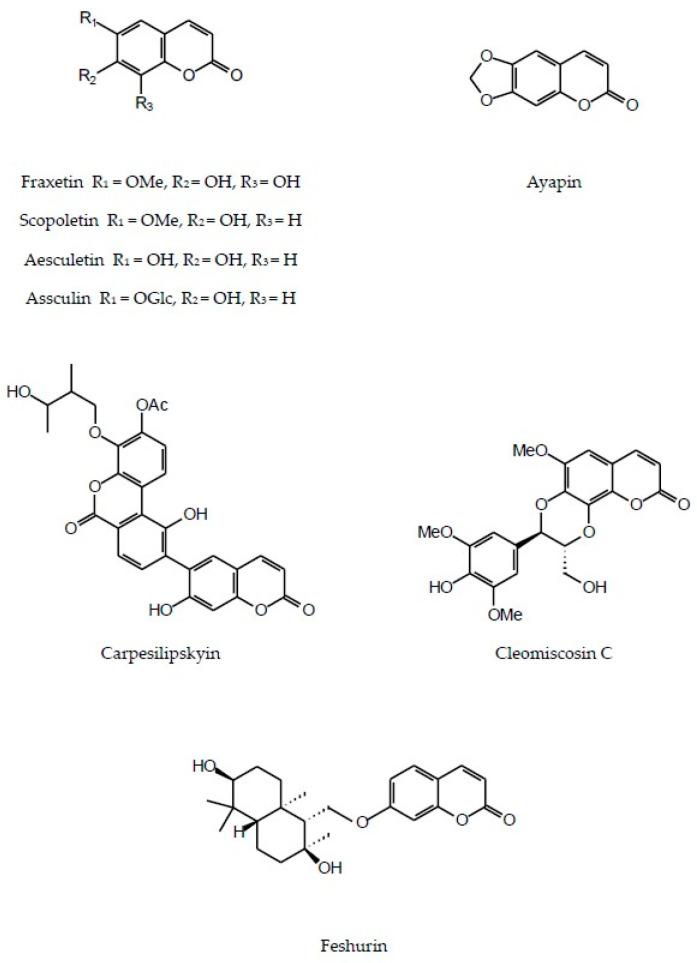
Structures of the selected coumarins from the Inulae-Inulinae.

Like other plants of the Asteraceae family, Inuleae-Inulinae accumulated mainly simple coumarins (coumarin, umbelliferone, herniarin, esculetin, scopoletin, isoscopoletin, scoparone and others) (for structures, see Figure 7). The compounds may reduce the glucose absorption rate, increase the level of insulin, increase the cellular uptake of glucose or reduce the gluconeogenesis [346] and act as antitumor agents through different mechanisms [347]. Their pharmacological activity has been briefly summarized by Keri et al. [348] in their review of the anticonvulsant properties of coumarin derivatives.

Coumarins are usually minor constituents of the Inuleae-Inulinae. They were found in *Blumea* spp., *Carpesium* spp., *Chiliadenus* spp., *Dittrichia* spp., *Duhaldea* spp., *Inula* spp., *Pentanema* spp., *Pulicaria* spp. and *Rhanterium* spp. The most frequently isolated compound from this group was scopoletin [68,113,178,237,252,349,350,351]. Other coumarins were mostly detected in plant extracts by different analytical methods.

Ceylan et al. [40] detected coumarin in eight of the eleven extracts from different *Inula* and *Pentanema* species (*I. anatolica*, *I. discoidea*, *I. inuloides*, *I. peacockiana*, *I. sechmenii*, *I. thapsoides*, *I. viscidula*, *P. britannicum*). Coumarin was a minor component in the analyzed samples and its content did not exceed 0.018 mg per 1 g of the dry extract. In the roots of *I. grandiflora*, from a location in the Himalayas, the compound was one of the major phenolic metabolites detected (over 6 μg per 1 g of the dry root) [114]. Umbelliferone (7-hydroxycoumarin; hydrangin; skimmetin) was isolated from a chloroform fraction of the *P. gnaphalodes* extract [352] and tentatively identified in the extracts from roots and rhizomes of *D. nervosa* [201], *I. helenium* and *I. racemosa* [278]. Another simple coumarin, herniarin (7-methoxycoumarin), and its derivative 7-hydroxycoumarin-sesquiterpene ether (feshurin, see Figure 7) were the coumarins isolated from *P. gnaphalodes* in addition to umbelliferone [352]. Esculetin (6,7-dihydroxycoumarin; cichorigenin) was obtained from *C. lipskyi* [350], *P. dysenterica* [81] and *P. insignis* [94]. The compound and its 6-*O*-glucoside (aesculin) were also detected (by TLC) in *I. koelzii*, *I. stewartii* and *I. rhizocephala* [65]. Zhang et al. [178], from the whole plants of *I. hookeri,* isolated a derivative of esculetin, ayapin (6,7-methylenedioxycoumarin). Scopoletin (6-methoxyesculetin; gelsemic acid; 6-methoxy-7-hydroxycoumarin) was obtained from the plants of the genera *Carpesium* (*C. lipskyi*, *C. macrocephalum*) [349,350] *Chiliadenus* (*C. candicans*) [252], *Duhaldea* (*D. cappa*, *D. wissmanniana*) [68,113], *Inula* (*I. hookeri*) [178] and *Pulicaria* (*P. burchardii*) [237] and was identified (by GC-MS) in the extract from *R. epapposum* [351]. The compound (from *C. macrocephalum*) turned out to be devoid of antibacterial activity [349]. Scopolin (scopoletin 7-*O*-glucoside) was present in the extracts from *D. cappa* (0.13–0.22 mg/g) [68,126] and was tentatively identified in roots and rhizomes of *D. nervosa* [112,201], *I. helenium* and *I. racemosa* [278]. Isoscopoletin (6-hydroxy-7-methoxycoumarin; 7-methoxyesculetin) was found in *D. cappa* [68] and *D. nervosa* [112]. Scoparone (6,7-dimethoxycoumarin; 6,7-dimethylesculetin) was isolated from *C. candicans* [252] and identified in *P. undulata* (LC-MS) [120] and *P. glutinosa* (GC-MS) [353]. Hydrangetin (7-hydroxy-8-methoxycoumarin) was tentatively identified in *B. balsamifera* by Pang and coworkers [136]. Aerial parts of *P. wightiana* yielded 7,8-dihydroxy-6-methoxycoumarin (fraxetin) [247]. The compound was also provisionally identified in *B. balsamifera* [132] and *D. nervosa* [201]. Brahmi-Chendouh and coworkers [172] tentatively identified 3,7-dihydroxycoumarin during the LC-MS analysis of the deterpenated and defatted *D. viscosa* leaves.

Derivatives of simple coumarins with a higher molecular weight rarely occur in Inuleae-Inulinae. Cleomiscosin C (aquillochin), a derivative of fraxetin, was isolated from flowers of *D. cappa* [78] and a coumarin dimer carpesilipskyin was extracted from the aerial parts of *C. lipskyi* [350]. 

A compound of unusual structure, 6-hydroxycoumarin lauryl ether, isolated from *P. britannicum* proved to be inactive as an sEH inhibitor in contrast to 6,8-dihydroxycoumarin (IC_50_ = 26.93 μM), investigated in the same study [102].

### 2.7. Stilbenoids

**Figure 8:**
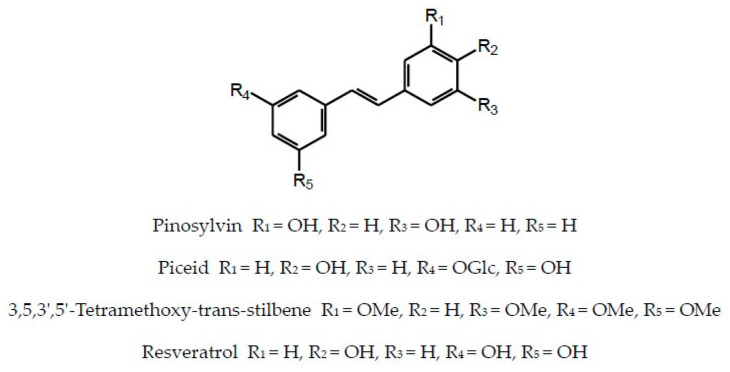
Structures of stilbenoids from Inulae-Inulinae.

Derivatives of stilbene may exert positive effects on the cardiovascular system and blood glucose levels. Their pharmacological properties, potential use in the therapy and mechanisms of action have been summarized by Dvorakova and Landa [354], Koh et al. [355] and Duta-Bratu et al. [356]. Only one compound from this class, pinosylvin (trans-3,5-dihydroxystilbene, see Figure 8) was isolated from the plants of the Inuleae-Inulinae. The stilbenoid was found in the aerial parts of *P. germanicum* by Bohlmann and coworkers [357]. Ceylan et al. [40] detected 3,4,5-trihydroxystilbene-3-*O*-glucoside (piceid) in three species from the genus *Inula* (*I. viscidula*, *I. inuloides* and *I. peacockiana*). The highest content of the compound was found in *I. viscidula* (0.07 mg per 1 g of the dry extract) [40]. 3,5,3′,5′-Tetramethoxy-trans-stilbene was tentatively identified in the extracts from roots and rhizomes of *I. helenium* and *I. racemosa*, ingredients of “tumuxiang”, a preparation used by TCM practitioners [280]. Traces of resveratrol were detected in the root extract from *C. montanum* [64].

### 2.8. Miscellaneous Compounds

Only three papers described phenylethanoids as metabolites of the Inuleae-Inulinae. Olennikov and Thankhaeva [335] isolated echinacoside from the roots of *I. helenium*. Chelly and coworkers [60] quantified phenolic metabolites, including phenylethanoids, in the methanol extract (yield 26.1%) from aerial parts of *R. suaveolens*. The extract contained verbascoside (1095 mg/100 g), oleuropein (260 mg/100 g), tyrosol (390 mg/100 g) and hydroxytyrosol (65 mg/100 g). For the comparison, the content of the major phenolic constituents of the extract, *p*-coumaric acid and apigenin 7-*O*-glucoside, reached 4540 mg/100 g and 4055 mg/100 g, respectively. Tubuloside A was tentatively identified in the extracts from the roots of *I. helenium* and *I. royleana* [280].

Traces of ellagic acid were detected in the extracts from the roots of *C. montanum* [64]. Tannic acid was found in four out of eleven *Inula* and *Pentanema* species investigated by Ceylan et al. [40]. Its content in the dry extracts ranged from 0.008 mg/g in *I. peacockiana* to 0.647 mg/g in *I. sechmenii*. Tannic acid was also detected and quantified in the leaves of *D. graveolens* [198]. However, the measured content of the compound was low (45 μg/kg of the dry extract).

Two enantiomers of britanicafanin A and britanicafanin B, polyphenols of the atypical structure, were isolated from *P. britannicum* as the active sEH inhibitors (IC_50_: 16.12 μM-24.05 μM) [102]. Gao et al., in the samples of “tumuxiang”, a preparation containing roots of *I. helenium* and/or *I. racemosa*, tentatively identified isomucronustyrene (CID: 10423261) and mulberrofuran A (CID: 5281332) [280].

## 3. Conclusions

Recently, a significant growth in the number of publications concerning phenolic metabolites of Inuleae-Inulinae has been observed. Introduction and popularization of the hyphenated analytical techniques (especially diverse variants of HPLC-MS) speeded up the process of uncovering compositions of plant extracts. However, the quality of the results obtained by the modern methods depends both on the quality of equipment and on the expertise of researchers. The published results were sometimes below the expectations. Our knowledge of the polyphenols produced and accumulated by the plants expanded rapidly thanks to the new methods, but there are still a lot of gaps to fill. Replacement of the time-consuming process of the isolation and spectroscopic analysis of plant metabolites by a single-step chromatographic analysis of the plant extract is tempting but still impossible because some structural details can not be resolved using LC-MS. The hyphenated methods, however, are indispensable as dereplication tools and may reveal the presence of compounds that are lost during the traditional analysis. Their potential to quantify the components of the pharmacologically active plant preparations seems to be underutilized.

Flavonoids are the most frequently investigated polyphenolic metabolites of the Inuleae-Inulinae. The compounds, however, do not dominate the polyphenolic metabolite fraction of every species included in the subtribe. *Blumea balsamifera* and *Dittrichia viscosa* seem to be especially rich in flavonoids of diverse structural types whereas only nine flavonoids were described as metabolites of *Carpesium* spp. Some of the flavonoids isolated from the Inuleae-Inulinae demonstrated cytotoxic activity towards human cancer cell lines in vitro. The anticancer activity in some instances was confirmed in vivo, using transplantable tumor systems. The molecular mechanisms behind the selective cytotoxicity of the plant constituents against the cancer cells have only in part been elucidated. 

Hydroxycinnamates are the second most frequently studied group of polyphenols synthesized by the plants of the subtribe. Both flavonoids and hydroxycinnamates have been frequently tested in vitro for their antioxidative and anti-inflammatory properties with positive outcomes. Moreover, pharmacological research on the Inuleae-Inulinae polyphenols brought some interesting results, including those concerning blood glucose level and blood pressure lowering, adipogenesis regulation and counteracting depressive-like behavior. The results supported the concept that polyphenols participate in pharmacological effects exerted by the examined plant extracts. Studies on the hepatoprotective activity of the polyphenols and their lung injury protective effect are also worth noting. Taking into consideration the results achieved in vitro, the inhibitory activity of polyphenols towards the soluble epoxide hydrolase in living cells may be an interesting area to explore.

To sum up, the Inuleae-Inulinae subtribe of the Asteraceae comprises the plants that are producers of structurally diverse pharmacologically active polyphenols. Their therapeutic potential and molecular mechanisms of action have not yet been fully explored. To improve the quality of research and applicability of the results, pharmacological investigations of the plant extracts should be accompanied by the qualitative and quantitative analysis of the plant preparation used.

## Figures and Tables

**Table 1:** Commonly used plant binominal names and the current taxonomic nomenclature in the Inuleae-Inulinae subtribe.

Commonly Used Name	Current Botanical Nomenclature (According to WFO)
*Allagopappus dichotomus* subsp. *dichotomus*	*Allagopappus canariensis* (Willd.) Greuter
*Allagopappus dichotomus* Cass.	*Allagopappus canariensis* (Willd.) Greuter
*Anvillea radiata* Coss. and Durieu	*Anvillea garcinii* subsp. *radiata* (Coss. and Durieu) Anderb.
*Asteriscus maritimus* Less.	*Pallenis maritima* subsp. *maritima*
*Asteriscus pygmaeus* Coss. and Durieu	*Pallenis hierochuntica* (Michon) Greuter
*Blumea gariepina* DC.	*Laggera decurrens* (Vahl) Hepper and J.R.I.Wood
*Blumea glomerata* DC.	*Blumea fistulosa* Kurz
*Blumea laciniata* DC.	*Blumea sinuata* (Lour.) Merr.
*Inula aschersoniana* Janka	*Pentanema aschersonianum* (Janka) D.Gut.Larr., Santos-Vicente, Anderb., E.Rico and M.M.Mart.Ort.
*Inula bifrons* L.	*Pentanema bifrons* (L.) D.Gut.Larr., Santos-Vicente, Anderb., E.Rico and M.M.Mart.Ort.
*Inula britannica* L.	*Pentanema britannicum* (L.) D.Gut.Larr., Santos-Vicente, Anderb., E.Rico and M.M.Mart.Ort.
*Inula britannica* var. *chinensis* (Rupr. ex Maxim.) Regel	*Inula japonica* Thunb.
*Inula britannica* var. *japonica* (Thunb.) Franch. and Sav.	*Inula japonica* Thunb.
*Inula cappa* (Buch.-Ham. ex D.Don) DC	*Duhaldea cappa* (Buch.-Ham. ex D.Don) Pruski and Anderb.
*Inula conyza* DC.; *Inula conyzae* (Griess.) Meikle	*Pentanema conyzae* (Griess.) D.Gut.Larr., Santos-Vicente, Anderb., E.Rico and M.M.Mart.Ort.
*Inula crithmoides* L.	*Limbarda crithmoides* (L.) Dumort.
*Inula ensifolia* L.	*Pentanema ensifolium* (L.) D.Gut.Larr., Santos-Vicente, Anderb., E.Rico and M.M.Mart.Ort.
*Inula falconeri* Hook.f.	*Pentanema caspicum* (F.K.Blum ex Ledeb.) G.V.Boiko, Korniy. and Mosyakin
*Inula germanica* L.; *Inula orientalis* Willd.	*Pentanema germanicum* (L.) D.Gut.Larr., Santos-Vicente, Anderb., E.Rico and M.M.Mart.Ort.
*Inula grantioides* Boiss.	*Iphiona grantioides* (Boiss.) Anderb.
*Inula graveolens* (L.) Desf.	*Dittrichia graveolens* (L.) Greuter
*Inula mariae* Bordz.	*Pentanema mariae* (Bordz.) D.Gut.Larr., Santos-Vicente, Anderb., E.Rico and M.M.Mart.Ort.
*Inula montana* L.	*Pentanema montanum* (L.) D.Gut.Larr., Santos-Vicente, Anderb., E.Rico and M.M.Mart.Ort.
*Inula nervosa* Wall.	*Duhaldea nervosa* (Wall. ex DC.) Anderb.
*Inula oculus-christi* L.; *Inula montana* M.Bieb.	*Pentanema oculus-christi* (L.) D.Gut.Larr., Santos-Vicente, Anderb., E.Rico and M.M.Mart.Ort.
*Inula orientalis* Lam.	*Pentanema orientale* (Lam.) D.Gut.Larr., Santos-Vicente, Anderb., E.Rico and M.M.Mart.Ort.
*Inula royleana* C.B.Clarke	*Inula racemosa* Hook.f.
*Inula salicina* L.	*Pentanema salicinum* (L.) D.Gut.Larr., Santos-Vicente, Anderb., E.Rico and M.M.Mart.Ort.
*Inula spiraeifolia* L.; *Inula germanica* Vill.	*Pentanema spiraeifolium* (L.) D.Gut.Larr., Santos-Vicente, Anderb., E.Rico and M.M.Mart.Ort.
*Inula viscosa* (L.) Aiton	*Dittrichia viscosa* subsp. *viscosa*-*Dittrichia viscosa* (L.) Greuter
*Inula wissmanniana* Hand.-Mazz.	*Duhaldea wissmanniana* (Hand.-Mazz.) Anderb.
*Jasonia candicans* (Delile) Botsch.	*Chiliadenus candicans* (Delile) Brullo
*Jasonia glutinosa* (L.) DC.	*Chiliadenus glutinosus* Fourr.
*Jasonia montana* (Vahl) Botsch.	*Chiliadenus montanus* (Vahl) Brullo
*Nauplius aquaticus* Cass.	*Asteriscus aquaticus* (L.) Less.
*Pentanema divaricatum* Cass.	*Vicoa divaricata* (Cass.) Oliv. and Hiern
*Pentanema glanduligerum* (Krasch.) Gorschk.	*Vicoa glanduligera* Krasch.
*Pentanema indicum* (L.) Y.Ling	*Vicoa indica* DC.
*Pulicaria crispa* Forssk. Benth. et Hook. f; *Francoeuria crispa* (Forssk.) Cass.	*Pulicaria undulata* (L.) C.A.Mey. including *P. undulata* subsp. *undulata*
*Varthemia candicans* Boiss.	*Chiliadenus candicans* (Delile) Brullo
*Varthemia iphionoides* Boiss. and C.I.Blanche	*Chiliadenus iphionoides* (Boiss. and C.I.Blanche) Brullo
*Xerolekia speciosissima* (L.) Anderb.	*Buphthalmum speciosissimum* L.

## Data Availability

Not applicable.
